# Landscape-level human disturbance results in loss and contraction of mammalian populations in tropical forests

**DOI:** 10.1371/journal.pbio.3002976

**Published:** 2025-02-13

**Authors:** Ilaria Greco, Lydia Beaudrot, Chris Sutherland, Simone Tenan, Chia Hsieh, Daniel Gorczynski, Douglas Sheil, Jedediah Brodie, Mohammad Firoz Ahmed, Jorge Ahumada, Rajan Amin, Megan Baker-Watton, Ramie Husneara Begum, Francesco Bisi, Robert Bitariho, Ahimsa Campos-Arceiz, Elildo A. R. Carvalho, Daniel Cornélis, Giacomo Cremonesi, Virgínia Londe de Camargos, Iariaella Elimanantsoa, Santiago Espinosa, Adeline Fayolle, Davy Fonteyn, Abishek Harihar, Harry Hilser, Alys Granados, Patrick A. Jansen, Jayasilan Mohd-Azlan, Caspian Johnson, Steig Johnson, Dipankar Lahkar, Marcela Guimarães Moreira Lima, Matthew Scott Luskin, Marcelo Magioli, Emanuel H. Martin, Adriano Martinoli, Ronaldo Gonçalves Morato, Badru Mugerwa, Lain E. Pardo, Julia Salvador, Fernanda Santos, Cédric Vermeulen, Patricia C. Wright, Francesco Rovero

**Affiliations:** 1 Department of Biology, University of Florence, Florence, Italy; 2 Department of Integrative Biology, Michigan State University, Michigan, United States of America; 3 Ecology, Evolution, and Behavior Program, Michigan State University, Michigan, United States of America; 4 Centre for Research into Ecological and Environmental Modelling, University of St Andrews, St Andrews, United Kingdom; 5 National Research Council, Institute of BioEconomy (CNR-IBE), San Michele all’Adige, Italy; 6 Division of Biological Sciences, University of Montana, Missoula, Montana, United States of America; 7 Department of Environmental Sciences, Wageningen University and Research, Wageningen, the Netherlands; 8 Center for International Forestry Research (CIFOR), Kota Bogor, Jawa, Barat, Indonesia; 9 Faculty of Environmental Sciences and Natural Resource Management, Norwegian University of Life Sciences, Ås, Norway; 10 Division of Biological Sciences and Wildlife Biology Program, University of Montana, Missoula Montana, United States of America; 11 Institute of Biodiversity and Environmental Conservation, Universiti Malaysia Sarawak, Kota Samarahan, Sarawak, Malaysia; 12 Aaranyak, 13, Tayab ali Byelane, Bishnu Rabha Path, Guwahati, Assam, India; 13 Moore Center for Science, Conservation International, Arlington, Virginia, United States of America; 14 Zoological Society of London, Regents Park, London, United Kingdom; 15 The Nature Conservancy, Arlington, Virginia, United States of America; 16 Department of Life Science and Bioinformatics, Assam University (Diphu Campus), Diphu, Karbi Anglong, Assam, India; 17 Environment Analysis and Management Unit, Guido Tosi Research Group, Department of Theoretical and Applied Sciences, Insubria University, Varese, Italy; 18 Institute of Tropical Forest Conservation, Mbarara University of Science and Technology, Kabale, Uganda; 19 Southeast Asia Biodiversity Research Institute, Chinese Academy of Sciences, Yunnan, China; 20 Center for Integrative Conservation, Xishuangbanna Tropical Botanical Garden, Chinese Academy of Sciences, Yunnan, China; 21 Centro Nacional de Pesquisa e Conservação de Mamíferos Carnívoros (CENAP), Instituto Chico Mendes de Conservação da Biodiversidade (ICMBio), Atibaia, SP, Brazil; 22 Cirad, Université Montpellier, UR Forests & Societies, Montpellier Cedex 5, France; 23 Istituto Oikos E.T.S., Milano, Italy; 24 RPPN Estação Veracel, Eunápolis, Bahia, Brazil; 25 Centre ValBio, Ranomafana, Ifanadiana, Madagascar; 26 Facultad de Ciencias, Universidad Autónoma de San Luis Potosí, San Luis Potosí, Mexico; 27 Escuela de Ciencias Biológicas, Pontificia Universidad Católica del Ecuador, Quito, Ecuador; 28 Forest is Life, Gembloux Agro-Bio Tech, University of Liège, Gembloux, Belgium; 29 Panthera, New York City, New York, United States of America; 30 Nature Conservation Foundation, Mysore, India; 31 The University of Exeter, Geography, College of Life and Environmental Sciences, Amory Building, Exeter, United Kingdom; 32 Department of Zoology & Biodiversity Research Centre, University of British Columbia, Vancouver, Canada; 33 Felidae Conservation Fund, Mill Valley California, United States of America; 34 Department of Environmental Sciences, Wageningen University and Research, Wageningen, the Netherlands; 35 Smithsonian Tropical Research Institute, Balboa, Ancon, Panama; 36 Department of Field Conservation and Science, Bristol Zoological Society, Bristol, United Kingdom; 37 Department of Anthropology and Archaeology, University of Calgary, Calgary, Canada; 38 Laboratório de Biogeografia da Conservação e Macroecologia, Instituto de Ciências Biológicas, Universidade Federal do Pará, Belém, Brazil; 39 School of the Environment, The University of Queensland, Brisbane, Queensland, Australia; 40 Centre for Biodiversity and Conservation Science, University of Queensland, St. Lucia, Queensland, Australia; 41 Instituto Pró-Carnívoros, Atibaia, Brazil; 42 Laboratório de Ecologia e Conservação (LAEC), Departamento de Biologia, Faculdade de Filosofia, Ciências e Letras de Ribeirão Preto (FFCLRP), Universidade de São Paulo, Ribeirã Preto, Brazil; 43 Department of Wildlife Management, College of African Wildlife Management, Mweka, Kibosho Mashariki, Moshi, Tanzania; 44 Department of Ecological Dynamics, Leibniz Institute for Zoo and Wildlife Research, Berlin, Germany; 45 Faculty VI–Planning Building Environment, Institute of Ecology, Technische Universität Berlinn, Berlin, Germany; 46 School of Natural Resource Management, George Campus, Nelson Mandela University, South Africa; 47 Grupo de Conservación y Manejo de Vida Silvestre, Universidad Nacional de Colombia, Bogotá, Colombia; 48 Department of Ecosystem Science and Management, Ecology and Evolution Program, University of Wyoming, Laramie, Wyoming, United States of America; 49 Departamento de Mastozoologia, Coordenação de Zoologia, Museu Paraense Emílio Goeldi, Belém Pará, Brazil; 50 Department of Anthropology, Stony Brook University, Stony Brook, New York, United States of America; 51 MUSE-Museo delle Scienze, Trento, Italy; Centre National de la Recherche Scientifique, FRANCE

## Abstract

Tropical forests hold most of Earth’s biodiversity and a higher concentration of threatened mammals than other biomes. As a result, some mammal species persist almost exclusively in protected areas, often within extensively transformed and heavily populated landscapes. Other species depend on remaining remote forested areas with sparse human populations. However, it remains unclear how mammalian communities in tropical forests respond to anthropogenic pressures in the broader landscape in which they are embedded. As governments commit to increasing the extent of global protected areas to prevent further biodiversity loss, identifying the landscape-level conditions supporting wildlife has become essential. Here, we assessed the relationship between mammal communities and anthropogenic threats in the broader landscape. We simultaneously modeled species richness and community occupancy as complementary metrics of community structure, using a state-of-the-art community model parameterized with a standardized pan-tropical data set of 239 mammal species from 37 forests across 3 continents. Forest loss and fragmentation within a 50-km buffer were associated with reduced occupancy in monitored communities, while species richness was unaffected by them. In contrast, landscape-scale human density was associated with reduced mammal richness but not occupancy, suggesting that sensitive species have been extirpated, while remaining taxa are relatively unaffected. Taken together, these results provide evidence of extinction filtering within tropical forests triggered by anthropogenic pressure occurring in the broader landscape. Therefore, existing and new reserves may not achieve the desired biodiversity outcomes without concurrent investment in addressing landscape-scale threats.

## Abstract 2

Tropical forests hold most of Earth’s biodiversity and a higher concentration of threatened mammals than other biomes. This study uses a standardized pan-tropical dataset of 239 mammal species from 37 forests across 3 continents to provide evidence of selective extinction occurring within tropical forests triggered by anthropogenic pressure in the broader landscape.

## Introduction

While the severity of the current biodiversity crisis is debated [1,2], defaunation, i.e., the loss of animals from local population declines, range contractions, and extinction, is widely documented [3,4]. Tropical forests represent the most species-rich terrestrial biome, yet human population growth and associated land conversion continue to make habitat loss, fragmentation, and unsustainable hunting major drivers of vertebrate decline [5,6], resulting in the highest concentration of threatened mammals relative to other biomes [4,7]. Some mammal species in tropical forests now survive almost exclusively in protected areas (hereafter PAs) [8] that often occur within extensively transformed and heavily populated landscapes; others depend on remote and sparsely populated areas [9,10]. However, it remains unclear how mammalian communities within tropical forest respond to pressures in the broader landscape in which they are embedded. Some evidence suggests that even biodiversity within PAs is threatened by higher anthropogenic threats outside their borders [10–13]. For example, forest loss in the 50 km surrounding tropical forest PAs contributed to a decline in tree species richness within PAs [11]; forest fragmentation in a 10 km buffer around monitoring sites reduced colonization rates of forest-specialist mammals [14]. Nevertheless, empirical evidence and assessments of the consequences of landscape-level human impacts on the richness and occupancy status of tropical forest wildlife communities are lacking. As governments commit to increasing the global PA extent to prevent further biodiversity loss following the Kunming-Montreal Global Biodiversity Framework [15], identifying the landscape-level conditions that can enhance wildlife conservation is paramount [16].

**Fig 1:**
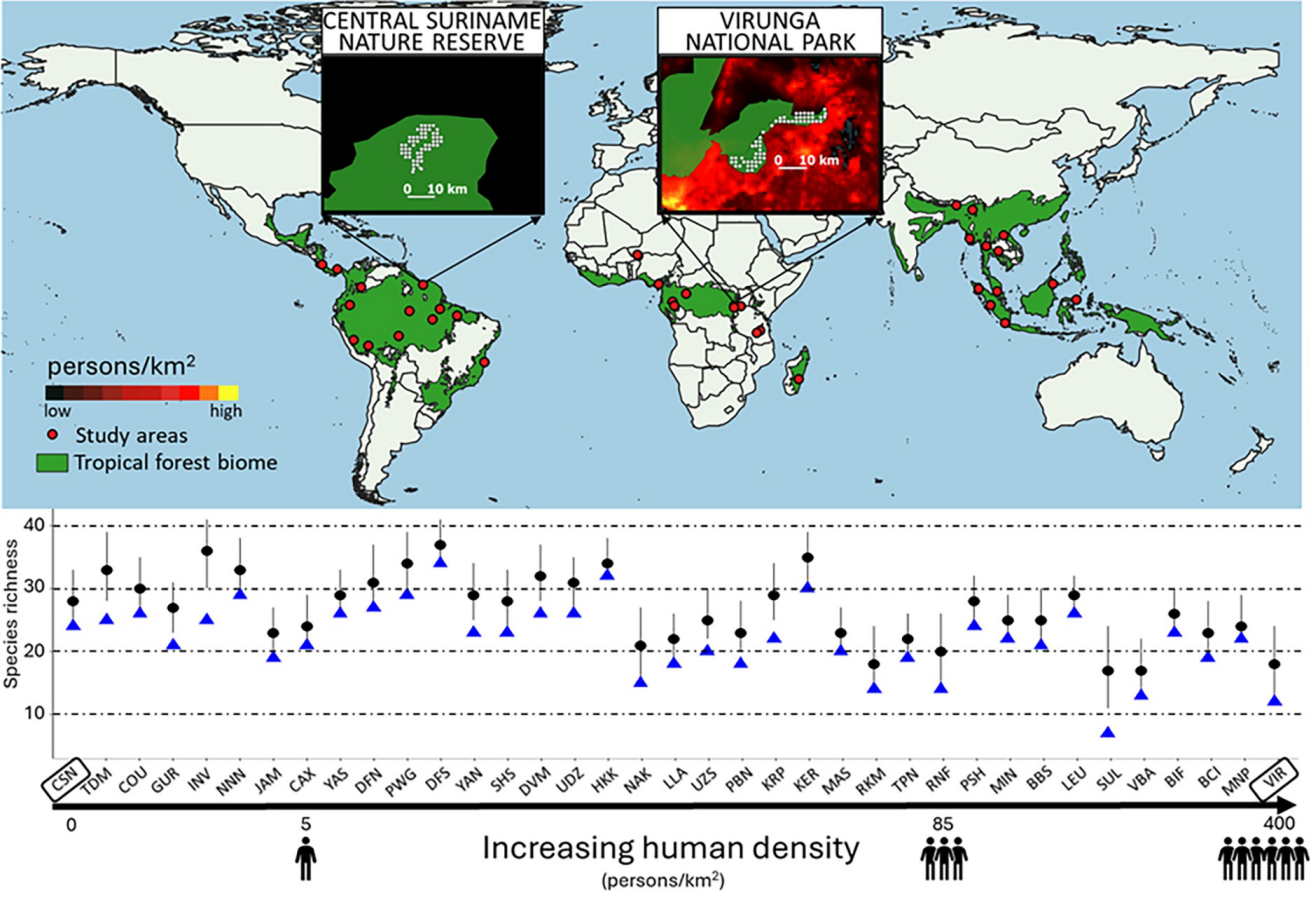
Map of the 37 tropical forest areas considered in the study. (A) Inset maps represent 2 protected areas embedded in contrasting landscapes: landscapes with low human density (left) and landscapes with high human density (right). Green areas in the inset maps represent PA boundaries. (B) Bottom chart shows estimated species richness (black dots) and the observed number of detected species with camera-traps (blue triangles) in each forest, which are ordered by increasing human density. See S4 Table in the SI Appendix for the list of site codes. Forest cover layer derived from Hansen and colleagues [102], whereas human population density layer derived from Gridded Human Population of the World [89]. The data underlying this figure can be found in S1 Data. PA, protected area.

To date, data quality, geographic coverage, and methodological inconsistencies have prevented rigorous objective examination of the status of tropical forest wildlife. Large-scale assessments of mammalian species richness are typically based on range maps (e.g., [17,18]), which can overestimate species occurrence [19] and lack information on abundance. For mobile species, range maps confound absence with non-detection, potentially biasing the estimation of true community size [20]. Literature-based meta-analyses are prone to additional biases due to heterogeneity in data quality and spatiotemporal scales [21]. In contrast, in situ data collected systematically at broader scales produce robust inferences that are more appropriate for evaluating trends and testing hypotheses at continental and global scales [22]. Camera-trapping has revolutionized wildlife monitoring [23], with its cost-effectiveness and unmatched potential for scaling-up and standardization [24]. Here, we collected an extensive tropical data set of detections for 239 primarily ground-dwelling mammalian species in 37 forests throughout the tropics collected from 2,021 camera-traps (Fig 1). Sampled areas included those forming the TEAM Network [25] plus 20 additional areas that extend the geographic coverage and the gradients of anthropogenic pressure and legal protection status. Sampled area landscapes ranged from nearly intact to severely fragmented and human-dominated landscapes, and legal protection status ranged from PAs to multiple-use and unmanaged forests. This newly compiled and standardized data set of wildlife data collected throughout the tropics provides a unique opportunity to quantify the effects of anthropogenic disturbance on mammal richness and occupancy throughout tropical forests globally.

**Fig 2:**
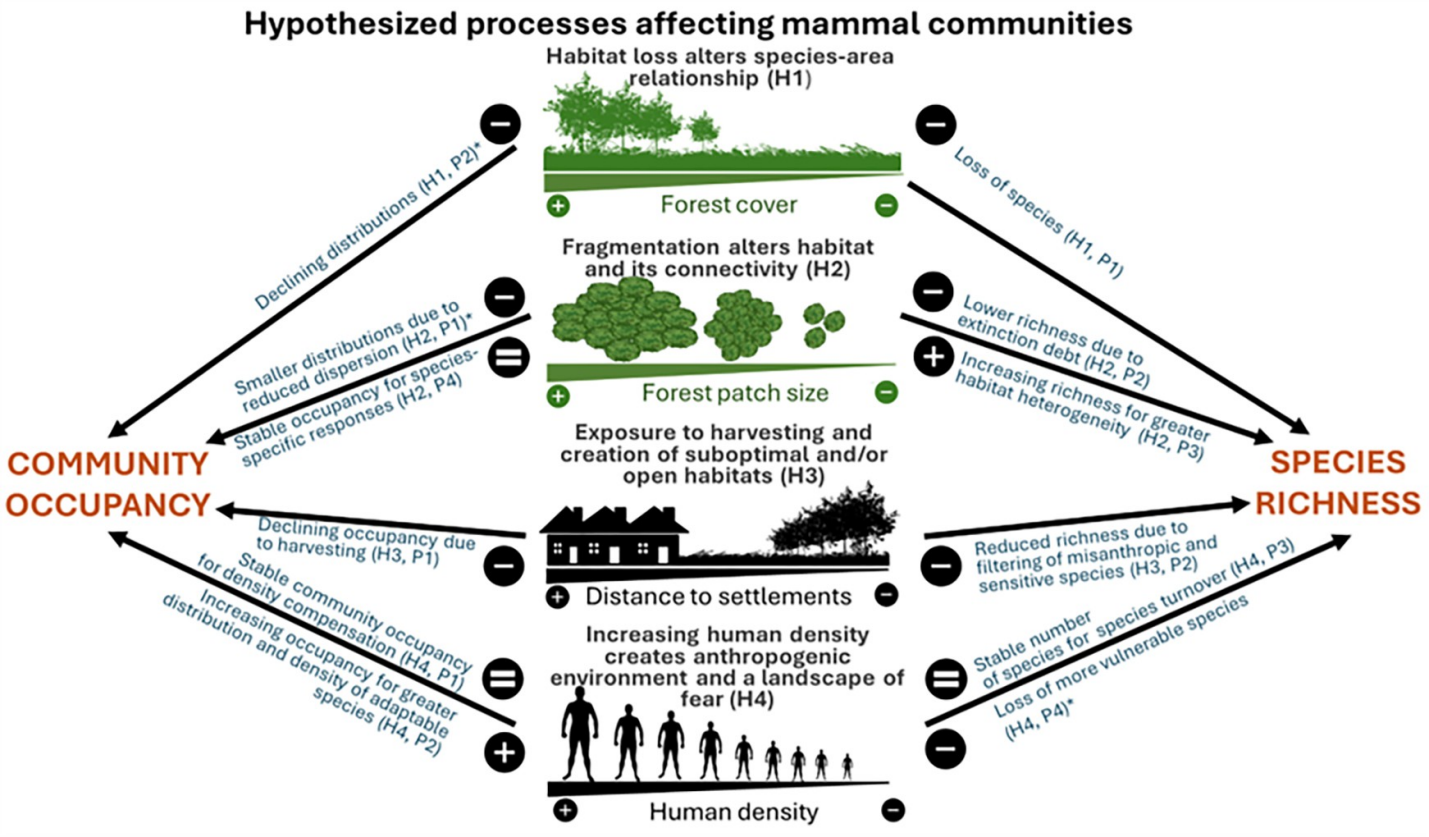
Hypothesized landscape-scale processes (H1– H4) that may influence the species richness and community occupancy of mammal communities. The figure depicts the hypothesized influence of landscape-scale forest cover (H1), forest configuration (i.e., fragmentation and connectivity of remnant forest patches) (H2), distance to settlements (H3), and human density (H4). Landscape is defined as the area extending 50 km from camera-trap arrays [11]. Asterisks indicate predictions supported by the results.

We test 4 non-mutually exclusive hypotheses for mechanisms linking variation in mammal community richness and occupancy to landscape-scale anthropogenic processes in tropical forests. Species richness represents the size of the mammalian community and here we focus on its geographic (among-area) variation, while community occupancy represents the central tendency of site use probability for all species belonging to a community. Occupancy is a measure of distribution [26,27] that accounts for imperfect detection and is a useful metric to overcome the inherent difficulties of abundance estimation [28]. It reflects site-level responses of wildlife to local pressures that can manifest potentially within a short time, and it ultimately affects species richness when a species no longer occupies an area. Hence, species richness and community occupancy provide complementary information on how mammalian communities respond to anthropogenic drivers of both local species extinction and population decline [29]. We test how they vary in response to (1) the amount of forest habitat loss; (2) degree of forest fragmentation; (3) distance to settlements; and (4) level of human density in the broader landscape (Fig 2) [11].

Our first hypothesis is that as more forest cover is lost, remaining forest patches become isolated islands unable to sustain the pristine array of species due to species-area effects and altered capacity to support viable populations. Hence, where landscape forest cover is lower, we predict lower mammalian richness (Hypothesis (H) 1, Prediction (P) 1). Although Haddad and colleagues [30] have found no overall effect of area reduction on abundance, we hypothesize lower community occupancy where landscape forest cover is lower (H1, P2). Indeed, before a local extinction occurs, a population declines in abundance with consequent shrinkage of its distribution, resulting in a decrease in species-level occupancy. Second, we hypothesize that the size and shape of remnant forest patches in the landscape (i.e., habitat configuration) can affect population source-sink dynamics among monitoring sites and surrounding landscapes [31]. Forest loss has resulted in 70% of remaining tropical forests occurring within 1 km from an edge [30]. Reduced patch area and increasing isolation have been associated with reduced abundances for a range of taxa, including mammals [30]. Similarly, global assessments have shown reduced mammal distributions with higher fragmentation [32]. Hence, we predict reduced mammalian community occupancy in landscapes with more fragmented forest cover (H2, P1). We also predict lower species richness with increasing fragmentation (H2, P2) as a result of relict populations disappearing over time as a manifestation of the extinction debt [33]. Conversely, fragmentation may create more habitat heterogeneity colonized by generalist species [34]; hence, we alternatively predict an increase in species richness (H2, P3). Additionally, anthropogenic edges can promote the use of open habitats and agricultural matrices for dietary resources or refugia by some species [35], which may result in a lack of variation in community occupancy in response to the degree of fragmentation (i.e., reduced patch size and increased patch isolation) as some species have higher occupancy near edges while others spatially avoid them (H2, P4).

Third, we hypothesize that the presence of settlements and infrastructure in the landscape favors human permeability into forest interiors, driving the exploitation of resources and hunting [36]. We, therefore, predict lower mammalian community occupancy near infrastructure (H3, P1). We also predict lower species richness as the potential outcome of human-induced filtering of sensitive and misanthropic species [37] (H3, P2). Fourth, we assume that higher landscape-scale human population density represents both a direct stressor to wildlife and a proxy for threats that are unknown or difficult to measure, such as demand for bushmeat products [38] and forest resource extraction, which may create a landscape of fear [39,40]. Human density can affect species presence and distribution, resulting in either range expansions or contractions for species that exploit anthropogenic environments or avoid them, respectively [39,41]. While predicting an outcome is difficult given human density can serve as a proxy for multiple complex processes, we test the following alternative predictions for both community metrics: (1) an increase in abundance of synanthropic species or species not targeted by hunters via density compensation or ecological release offsets the decline of sensitive species [42,43]. We therefore predict no net change in average community occupancy in response to human density (H4, P1). (2) Alternatively, as a positive relationship has been found between increasing mammal densities and high level of Human Footprint Index globally [44], we hypothesize an increase in community occupancy (H4, P2) as the result of domination by human-tolerant species. For species richness, (3) we predict no variation (H4, P3) as the local disappearance of sensitive species may be compensated for by the colonization of new adaptable ones [1,45]. However, for 16 tropical forest PAs, higher human density in the immediate surroundings was associated with lower richness of area-demanding and habitat specialist guilds, particularly insectivores [46]. In US parks, greater human density in a 50 to 100 km buffer predicted mammal extinction inside PAs [47]. Thus, (4) we alternatively predict lower mammalian richness with higher surrounding human density as the outcome of species filtering, with loss of sensitive and less adaptable species (H4, P4).

Coupling this newly synthesized data set with state-of-the-art community modeling, we simultaneously test hypotheses about factors contributing to geographic variation in the absolute size (species richness) and distribution (community occupancy) of partially observed ecological communities. We test hypotheses about variation at both levels of community structure in relation to forest cover, habitat configuration, distance to settlements, and human density measured throughout the camera-trap monitoring sites and a 50-km buffer surrounding them for each forest (Figs 2 and S1). We also include the size and habitat productivity of the sampled areas, as well as the continent and the coefficient of variation (CV) of annual rainfall, to account for their potential influences on species richness. To our knowledge, this is the first study that simultaneously models species richness and community occupancy across many areas at once as complementary metrics of community structure, providing for a novel and powerful approach to studying how tropical forest wildlife communities respond to landscape-scale drivers of change globally.

## Results

### Species richness

**Fig 3:**
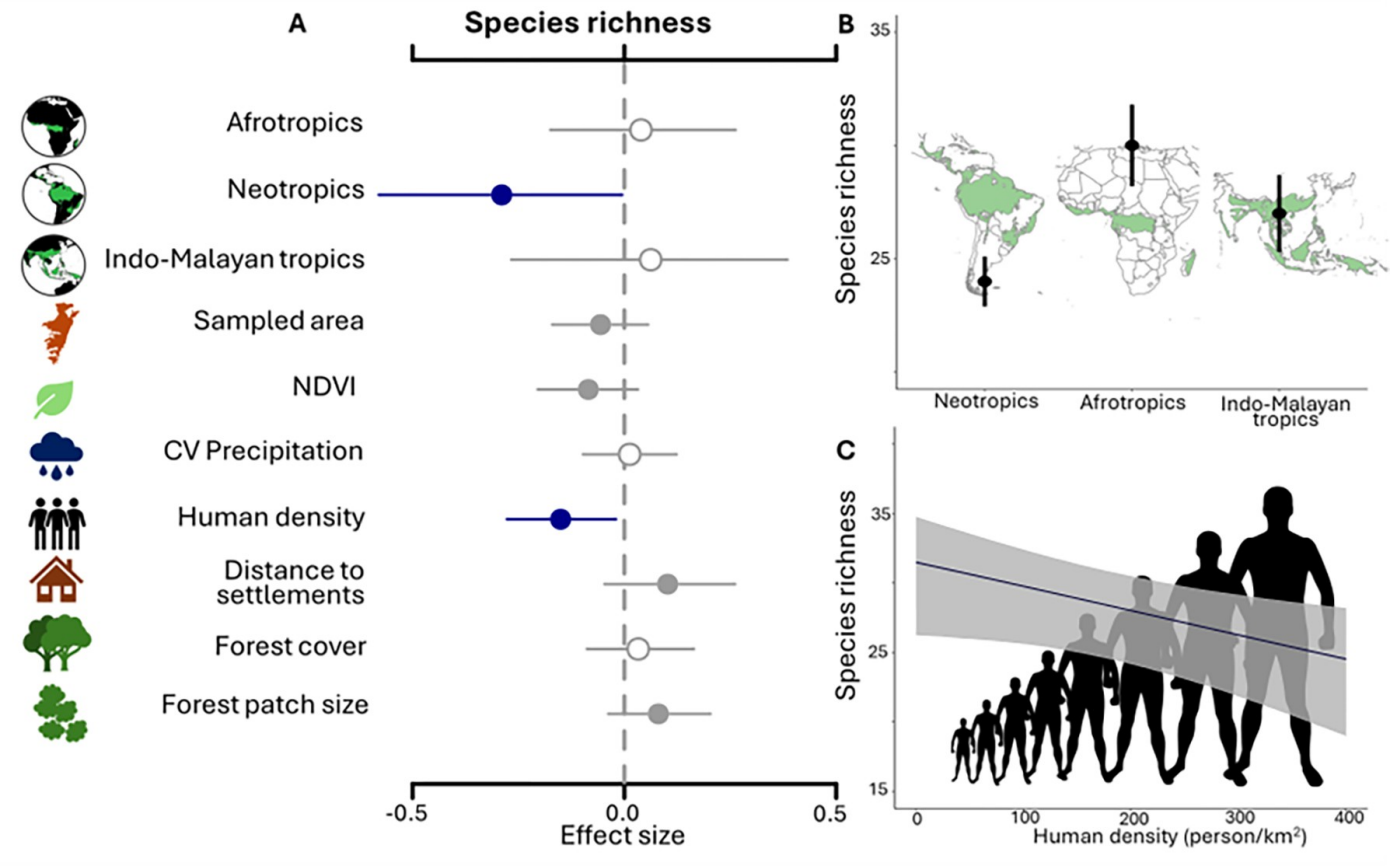
Predictors of mammalian species richness estimated from the multi-region, multispecies occupancy model using 37 study areas throughout the tropics. Standardized beta coefficients for the effects of predictors (A), with human density, forest cover, and forest patch size measured on a buffer extending 50 km from camera-trap arrays, whereas NDVI and CV precipitation were measured at the sampled area-level. Points indicate the median of the full posterior distribution. Solid blue lines represent significant effects with 90% Bayesian CI that do not overlap 0 (dashed vertical line). Closed gray points represent 50% CI that do not include 0, while open gray points indicate that 50% CI overlaps 0. Plots of predicted species richness depict patterns of species richness in relation to biogeographical area (B) and increasing human density in the broader landscape (C). Gray shaded areas indicate 90% CI. Forest cover layer for the 3 continents in panel B derived from Hansen and colleagues [102]. The data underlying this figure can be found in S2 Data. CI, credible interval; CV, coefficient of variation; NDVI, normalized difference vegetation index.

We focus first on factors that explain geographic variation in the size of mammalian communities, i.e., species richness. Estimated richness ranged from a low of 17 species in Volcan Barva, Costa Rica (90% Bayesian credible interval, CI: 14–22) and Sulawesi, Indonesia (CI: 11–24), to a high of 38 species (CI: 35–41) in Djoum, Cameroon (Fig 1). Species richness was significantly lower in the Neotropics (β = −0.29, CI: −0.60 – −0.01) than other continents (Fig 3 and S1 Table) and was significantly and negatively associated with landscape-scale human population density (β = −0.15; CI: −0.28 – −0.02). These results support the prediction that human pressure from higher population density in tropical forests negatively impacts species richness (H4, P4). Species richness was positively but not-significantly associated with greater forest patch size and with greater distance to settlements (Fig 3). In addition, mammalian richness was not significantly associated with greater forest cover, variation in annual precipitation, the size of the sampled area or habitat productivity (normalized difference vegetation index, NDVI) (Fig 3 and S1 Table).

### Community occupancy

**Fig 4:**
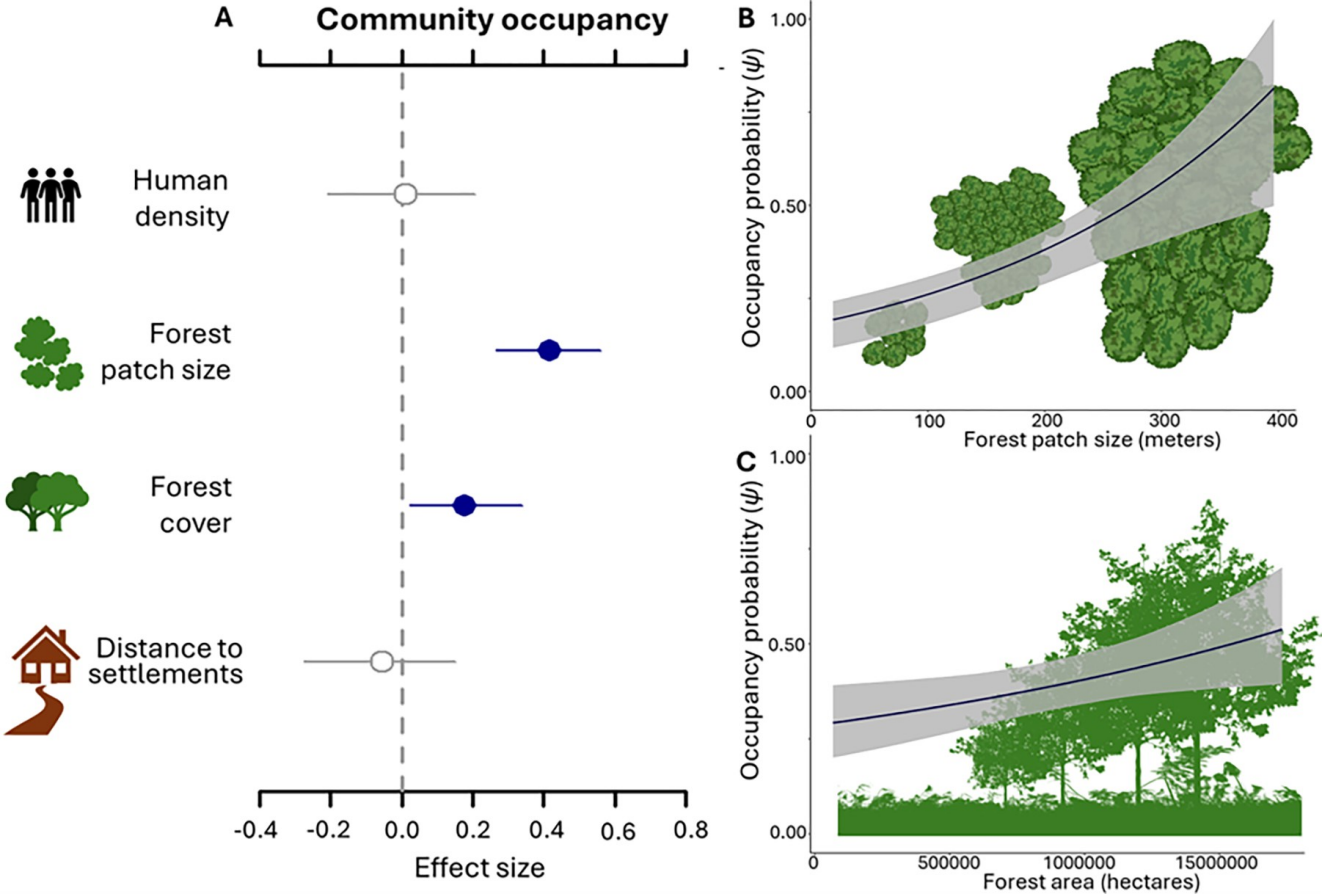
Predictors of community occupancy probability (*ψ*) estimated from the multi-region, multispecies occupancy model applied to 37 study areas throughout the tropics. Standardized coefficients for the effects of predictors (A). Human density, forest patch size, and forest cover were measured for a 50 km buffer from camera-trap arrays. Points indicate the median of the full posterior distribution. Solid blue lines represent significant effects with 90% Bayesian CI that do not overlap 0 (dashed vertical line). Closed gray points represent 50% CI that do not include 0, while open gray points indicate that 50% CI overlaps 0. Bivariate plots of the predicted community occupancy probability in relation to increasing forest patch size (B), and increasing forest cover (C). Gray shaded areas indicate 90% CI. The data underlying this figure can be found in S3 Data. CI, credible interval.

Next, we present drivers of global variation in community occupancy (i.e., average occupancy across the species assemblage), whereby lower occupancy suggests an overall shrinkage of the distribution and higher occupancy is indicative of a wider distribution. Compared to species richness, marked differences in the relationships with anthropogenic influences emerged. Occupancy was significantly and positively associated with higher landscape-scale forest cover (*θ* = 0.18; CI: 0.02–0.34) and with forest patch size (*θ* = 0.42; CI: 0.26–0.56; Fig 4). Hence, species within a community occupied a greater portion of the landscape where it had greater forest extent and consisted of larger and more continuous forest patches. Consequently, as predicted, habitat loss and fragmentation were negatively associated with community occupancy (H1, P1 and H2, P1, respectively). Unlike species richness, community occupancy was not associated with landscape-scale human density (H4, P1); similarly to richness, occupancy was not associated with distance to settlements (H3, P2; Fig 4). Overall, these results support the value and complementarity of the two scales of inference (i.e., species richness and community occupancy).

## Discussion

We empirically demonstrated the negative impacts of anthropogenic threats occurring in the broader landscape on both forest mammal species’ richness and community occupancy throughout the tropics. Previous attempts to address related questions using the TEAM camera-trap data focused exclusively on occupancy trends [48] or dynamics [14,49], functional composition [46,50], or taxonomic and functional diversity [51]. In contrast, our approach allowed for the simultaneous quantification of multiple drivers at 2 levels of community organization (species richness and community occupancy) while accounting for sampling biases, and extending both the number of areas and the gradient of anthropogenic disturbance in surrounding landscapes. Using standardized and systematic data recorded within tropical forests under varying degrees of anthropogenic disturbance, we provide much needed empirical evidence on hypothesized drivers of mammal community size and distribution throughout the tropics. Our results indicate that human density, habitat loss, and fragmentation have detrimental consequences for tropical forest wildlife worldwide. Specifically, we found that human density was the single, strongest predictor of species richness, whereby mammal communities occurring within areas surrounded by higher human population density had significantly fewer species (supporting H4, P4), even though most areas sampled were within PAs. Moreover, average mammal occupancy was lower in areas characterized by smaller and more fragmented areas of remaining forest in the landscape (versus larger and more continuous, supporting H1, P2 and H2, P1). Therefore, addressing the drivers impacting tropical forest wildlife critically depends on mitigating threats from human density and anthropogenic pressures that originate well beyond where populations occur [52].

That mammal species richness decreased as human population density in the landscape increased (hypothesis H4, P4 supported) is arguably the most robust empirical evidence to date documenting the detrimental effects of human presence on tropical wildlife. Specifically, our model predicts a 1% decline in species richness with an increase of 16 persons/km^2^ in the landscape. This result expands on the known negative effect of a sampling area’s human footprint on tropical mammal richness in Africa and Asia [51] to encapsulate impacts on species richness within tropical forests from well beyond sampling areas. High human density may lead to extirpation from overhunting [9] and species loss from other detrimental human activities [39]. Information on known local extinctions within recent decades independent of the camera-trap data analyzed was available for 11 of the monitored forests (S2 Table). The forests in this study where known local extinctions have occurred are among the areas under greatest anthropogenic pressure, including higher than average human density, further suggesting that contemporary anthropogenic threats have already impacted species richness. Across our data set, estimated species richness was among the lowest in Virunga National Park, Rwanda (18 species), surrounded by very high human population density (400 persons/km^2^) and a highly fragmented landscape. Indeed, the Virunga Massif has one of the highest human densities in the world [53]. In contrast, estimated species richness was among the highest in a sustainable logging concession in Gabon (PWG, 34 species), which is surrounded by very low human population (6.57 persons/km^2^). Furthermore, in the Udzungwa Mountains of Tanzania, the Udzungwa Scarp Nature Reserve has reduced mammal richness and occupancy when compared to the nearby Udzungwa Mountains National Park. Decades of isolation of the Nature Reserve’s forest, which has higher human density and more fragmented forest cover in the surrounding landscape relative to the National Park, led to the local extirpation of at least 3 species by the early 1970s, i.e., the buffalo (*Syncerus caffer*), the African elephant (*Loxodonta africana*), and the leopard (*Panthera pardus*) [54]. Surprisingly, we found that landscapes with more forest cover did not support richer communities of forest mammals, which contrasts with our hypothesis (H1, P1) and with the known positive relationship between the size of available habitat and species richness [55]. Additionally, species richness was not significantly associated with fragmentation, in contrast with H2, P2 and H2, P3. Rather than finding lower species richness in areas with greater habitat loss or fragmentation, the lack of significant effects may be due to turnover in species composition in modified environments, such as the replacement of forest-dependent species by habitat generalists that are able to colonize empty niches, resulting in a net-zero change in community size. Alternatively, the lack of a significant positive relationship between forest cover and species richness may suggest a degree of empty forest syndrome [56] in which forests remain but fauna have been lost for human disturbances other than habitat loss, such as hunting. We tested distance to settlements as a proxy for hunting because it reflects accessibility, but distance to settlement did not have a detectable effect on species richness. This result can be interpreted as a lack of evidence that accessibility reduced species richness or that the measure of distance to settlements may have been a poor hunting proxy.

In contrast to the negative effect of human density on species richness, human density did not predict variation in occupancy, supporting the prediction that high human density positively affects the distribution of some species while negatively affecting others, resulting in no net effect of human density on occupancy (supporting H4, P1). This density compensation mechanism may result from the net effect of negative human impact on misanthropic species and positive effect, via ecological release, on synanthropic species, or species not targeted by hunters. Such decoupling in the responses of richness and occupancy to human density may be a consequence of local extinction filtering, i.e., reduced distributions of the most vulnerable species and thriving of the more resilient ones [57]. The lack of support for higher community occupancy near high human density (H4, P2) does not preclude it from occurring if more sensitive species disappear over time under increasing pressure from human density. Critically moreover, mammal communities had higher occupancy when forest cover was more extensive (H1, P2) and less fragmented (H2, P1). Hence, larger and better-connected forest habitat sustains larger and more broadly distributed populations [58], whereas forest fragmentation contributes to lower mammal occupancy. That the effect of the landscape-scale habitat configuration on community occupancy was even stronger than that of forest cover may be attributable to fragmentation altering source-sink population dynamics (hypothesis H2, P1 supported), with a reduced capacity of some species to recolonize forest fragments after local populations are lost [59]. Interestingly, we note that while we addressed community-wide responses, the positive effects found in relation to both forest cover and forest patch size reveal that most species in the community benefit from increasing forest cover and patch size, even though these responses may be driven by the strict forest dependent species.

In contrast to our hypotheses, distance to settlements did not significantly predict variations in average community occupancy or species richness (hypothesis H3, P1 and H3, P2, rejected), suggesting that, similarly to the effect of human density on occupancy, infrastructure may positively affect the distribution and persistence of some species while negatively affecting others, resulting in no net effect [39,60]. While winners generally have faster life-history and more opportunistic diets when compared to losers [39,41], more work is required to determine which species characteristics best predict these outcomes [61]. Lastly, we found that landscape-scale anthropogenic pressures markedly affected species richness, while sampling area, precipitation, and NDVI did not, although larger sampling areas [55] and more productive forests [62] typically support more mammal species. This finding may reflect an adequate standardization of the camera-trap sampling design among areas while highlighting that landscape-scale anthropogenic impacts can override known ecological determinants of species richness such that the fundamental relationship between area and species richness was no longer detectable. In addition to the variables we examined, variation in richness among areas has likely been influenced by other factors, particularly biogeographic history [63]. In particular, the significantly lower richness estimated for the Neotropical fauna may reflect the disproportionally high Pleistocene extinctions, in which the continent lost ca 80% of large-bodied mammals [63].

By focusing on mammals in tropical forests, where similar environments support communities with similar functional composition and vulnerability to anthropogenic pressures [46,64], and by using in situ observational data from the largest number of sites and widest gradient of protection throughout the biome to date, we have shown that landscape-scale anthropogenic pressures are detrimental to wildlife, even within PAs. Indeed, 31 out of 37 sampled areas (84%) were within PAs, supporting earlier expert opinion-based evidence of their vulnerability to landscape threats [10]. For the 31 PAs, forest cover and forest patch size were significantly higher inside than outside PA borders (i.e., across the 50 km buffer; S2 Fig), further highlighting the role of landscape-scale habitat conditions for tropical mammals. The use of complementary metrics of community structure allowed us to determine that the effects of anthropogenic threats manifest differently for the total number of species supported compared to the distribution of the species present in the monitored forests. Our results demonstrate that the size and distribution of mammal communities in tropical forests are tied to human population density, forest cover loss, and degradation in the broader landscape where they occur.

Therefore, mammal conservation in tropical forests depends on mitigating the complex detrimental effects of anthropogenic pressures beyond PA borders [65–68]. Examples of such strategies include preventing further forest loss in the landscape, establishing buffer zones, and restoring habitat and connectivity in the wider matrix [68]. Most importantly, with half of the world population expected to reside in tropical regions by 2050 [6], our result of a marked impact of human density on wildlife indicates that holistic landscape-scale planning that harmonizes forest conservation with livelihood development becomes more imperative than ever. Multiple-use PAs and land-sharing approaches [68,69] are important strategies in this regard. Ninety percent of people living in extreme poverty in the tropics rely heavily on forest resources [70], thus improvement of living standards, promotion of alternatives to fuelwood for cooking and increased education can further allay tropical forest degradation [71]. In conclusion, our study warns that the creation of new PAs as provided by the Kunming-Montreal Global Biodiversity Framework may not achieve the desired biodiversity outcomes without concurrent investment in addressing landscape-scale threats. Towards this end, our results are relevant to the current UN Decade of Ecosystem Restoration [68], whereby priority locations for restoring tropical forests should be identified in landscapes surrounding isolated PAs, as these may promote persistence of tropical forests mammals.

## Methods

### Study areas

We collated data on 239 mammal species (S3 Table) from a standardized camera-trapping protocol replicated in 37 protected areas from 19 countries in 3 biogeographic regions throughout the tropical forest biome (Fig 1 and S2 Table): Afrotropics (*N* = 12), Neotropics (*N* = 13), and Indo-Malayan tropics (*N* = 12). Seventeen of the areas are from the TEAM Network [25]. We selected the additional camera-trap data sets on the remaining 20 areas based on adherence to the TEAM protocol (S1 and S3 Figs). Camera-trapping in TEAM areas followed a standardized protocol rolled out during one dry season (in 2014 or 2015), consisting of the systematic sampling of 60 camera-trap sites evenly spaced 1 to 2 km apart. In the additional areas, data were collected for a single-season during the period 2010 to 2019 with similar spacing among sites as TEAM’s and a range of 32 to 68 sites sampled (S5 Table). When effort varied, we selected the first 30 days of sampling. As a result, our data set collates camera-trap images from 37 areas, 2,021 camera-trap sites (mean of 54.76 per area), sampled through 63,041 camera-trap days (range 1,088 to 2,280, mean 1,703), and represents the largest standardized and coordinated sampling data for tropical mammals to date (S5 Table). We more than doubled the number of sampled areas of the TEAM Network and achieved a more balanced number of areas among continents and across anthropogenic gradients. In particular, we included 9 new areas in the Indo-Malayan tropics which were underrepresented in the TEAM data set (S3 Fig) despite Southeast Asia being a major hotspot of deforestation and biodiversity loss [72].

The additional areas extend the gradient of protection regime, anthropogenic disturbance, and landscape configuration relative to existing TEAM sites. Overall, the sampled forests include PAs of different status: from National Parks (*N* = 15) and Nature Reserves (*N* = 3) to PAs of lower protection regime (*N* = 13), from multiple-use (*N* = 2) to human-managed areas, as plantation and logging concession (*N* = 2), and forests with no legal protection status (*N* = 2, S4 Table). The landscape surrounding the study areas represented a wide range of human population density, from virtually unpopulated to highly populated (Fig 1), such that also well-protected PAs may suffer from intense external anthropogenic pressure.

For the analyses, we used 559,585 images of wild mammalian species with average body mass >100 g [48], as smaller species are not well detected by camera-traps and can be of difficult identification. We included in our analyses all species above this mass threshold and that are predominantly terrestrial, hence including species that are not all strictly forest dependent. Importantly, this restricts the targeted mammals to the medium-to-large ground dwelling species hence excluding small-bodied, strictly arboreal, and volant mammals. The targeted subset of species likely requires disproportionately larger areas and are more likely to be killed for food or from human–wildlife conflicts. We used IUCN taxonomy [73]. This resulted in 239 mammal species in 47 families, 17 orders, and 144 genera (S3 Table).

### Covariates description

For each study area, we derived a suite of 9 covariates, selected as a subset of candidate variables for the model based on removing collinearity. To identify potential multicollinearity, we used Pearson’s correlation coefficient with 0.6 as the maximum threshold (S4A Fig) and we considered the variance inflation factor (VIF), with values <4 acceptable (S4B Fig). Specifically, we considered covariates that broadly captured representative variation in habitat features, human disturbance, and sampling protocol. Habitat variables included: (1) forest cover in a buffer extending 50 km from the camera-trap arrays (range: 66,840–1,732,229 ha), as a measure of available forest habitat, with more forest habitat assumed to support higher biological diversity and richness [74,75]; (2) forest patch size, or forest compactness, in a buffer extending 50 km from the camera-trap arrays (range: 18.58–396.03 m) and representing the average distance one can move in a random direction within the patch, as a measure of decreasing fragmentation and hence greater connectivity [76,77]; (3) the normalized difference vegetation index (NDVI, range: 4,359–8,240) as an indicator of vegetation biomass and environmental productivity in the sampled area [78]; (4) the coefficient of variation of annual precipitation (range: 0.91–29.13) as an index of inter-annual climate variability. Human disturbance variables included: (5) distance from camera-trap sites to the closest settlement (range: 762–53,204 m), representing distance to settlements, forest accessibility to humans [36], and potential higher vulnerability to hunting pressure [79]; (6) the human population density in a buffer extending 50 km from the camera-trap arrays (range: 0.05–400.18 people/km^2^), as a measure of human presence and its impact on the landscape [47,80,81]. Sampling protocol variables included: (7) camera-trap trigger speed, given different camera models can perform differently in detecting species, especially elusive ones [82]; (8) sampled area (range: 55–168 km^2^) as an adjustment term for the different sampling effort across forests; (9) continents (i.e., Afrotropics, Neotropics, Indo-Malayan tropics) to account for the different locations and their evolutionary-history and phylogeny of different faunal groups [83]. We standardized all continuous covariates to have mean zero and unit standard deviation.

The covariates selected were extracted as follows:

1. Forest cover was estimated as the sum of forest cover patches within a buffer extending 50 km from the camera-trap arrays. It was computed using the R package “landscapemetrics” [84], with forest cover data set obtained from the Global Forest Cover [85] available in the R package “gfcanalysis” [86].
2. Forest patch size and compactness was estimated as the averaged radius of gyration of all forest patches within a buffer extending 50 km from the camera-trap arrays, where a greater mean radius coincides with greater patch size. The variable was extracted using the same R package and forest cover data set used for the previous variable.
3. NDVI was calculated by averaging the cell values of the 16-days MODIS NDVI images (L3 Global 250 m). Data acquisition in each study forest matched the year in which wildlife was sampled.
4. Coefficient of variation of annual precipitation describes the overall variability of rainfall recorded for an area. It was calculated as the standard deviation of precipitation from 2009 to 2018 divided by the mean annual precipitation over the same period [87]. This value was calculated at the centroid of each monitoring area.
5. Distance to the closest settlement was calculated using the global built-up surface layer from the Human Settlement layer at 250 m resolution from 2015 [88], derived from Landsat images to depict infrastructure of inhabited settlements. Specifically, we computed the Euclidean distance between each camera-trap station and the nearest vectorized cell with value > 0 of this layer, thus representing settlements from the size of hamlets or small villages. The distance was collinear with the distance to the closest roads (r = 0.71, *P* < 0.001); hence, the variable used in the analyses (i.e., distance to settlements) is intended to reflect both settlements and roads. Camera-level distances within each study area were averaged to obtain an area-level value.
6. Human density was calculated by averaging the cell values of the Gridded Population of the World raster [89] within a buffer extending 50 km from the camera-trap arrays. Data extraction was carried out using the R package “raster” [90].
7. Camera-trap trigger speed (1 = fast, 2 = moderate) quantified the trigger speed of camera models (i.e., the time from when the animal enters the field of view to when the camera takes a picture). Camera models were denoted as fast when the trigger speed was ca. 0.2 s (i.e., Reconyx and Panthera), while the other brands were denoted as moderate as trigger speed ranged from 0.25 to 1 s.
8. Sampled area was measured by creating and summing a buffer of 1 km around each camera-trap station.
9. Biogeographic regions (i.e., Afrotropics, Neotropics, Indo-Malayan tropics) were assigned according to the location of each study forest.

Polygons of the 1 km buffers around each camera-trap station (sampled area) and of the 50 km buffers around camera-trap arrays were created with the built-in tools in QGis [91].

### Statistical analysis

To test global-scale patterns of defaunation, we modeled the effect of area-specific covariates on species richness and community occupancy in the 37 forests using a multi-region community model that largely follows Sutherland and colleagues [92]. The 3 features of this hierarchical model are as follows. First, a sub model for species-specific detection allows for the fact that not all species are detected—this is achieved using a Bayesian technique called data augmentation [93]. Second, a sub model for occupancy: accounting for imperfect detection also allows for inferences to be made about the proportion of each area occupied by each of the species (observed and unobserved). We treat these species-specific occupancy rates as random effects from a community distribution. Thus, the mean of the community random effects distribution is modeled as a function of covariates (see above), i.e., the “community occupancy” rate. Notably, treating species-specific occupancy as a random effect provides the statistical flexibility to capture any within-region variation in site-specific occupancy. However, we did not test within-area variation in community occupancy with the covariates of interest because we were interested in its global scale patterns to test our hypotheses. Third, a sub model for the latent species richness, i.e., a linear model that relates area-specific covariates to the overall number of species in the area. The models for community occupancy and species richness were developed based on ecological a priori justifications and predictions [94,95].

Specifically, for computing the multi-region community model on R = 37 forests (hereafter regions) across the tropics, encounter frequencies of detected species were organized as a 3D array ***Y***, with elements *y*_*ijr*_ representing the number of days species *i* was observed at site *j* in region *r*, out of a total of *K*_*jr*_ days that the camera was operational. True occupancy states array ***Z*** contains elements *z*_*ijr*_ representing the species-by-site occupancy states during each survey, conditional on the species being a member of the community in each region (see below). Occupancy states were defined as Bernoulli random variables, with *z*_*ijr*_ = 1 indicating the site *j* is occupied by species *i*, and *z*_*ijr*_ = 0 if the site is empty: *z*_*ijr*_ ~ Bern (*ψ*_*ir*_ *ω*_*ir*_). Parameter *ω*_*ir*_ is a species-specific indicator variable denoting whether the species is present in a region, and *ψ*_*ir*_ is the species-specific occupancy probability in each region (assumed equal across sites), conditional on species *i* being a member of the *r*^*th*^ community. Based on data augmentation [93], an arbitrary number *Mr = M = 55* (for all regions) of all-zero encounter frequencies, which can be seen as potentially unobserved species, was added to the detection array ***Y***. The number of unobserved species in each community can therefore be estimated by evaluating which of the *M—n*_*r*_ species (rows of the augmented data set, where *n_r_* is the number of species detected in a region) are members of the *r*^*th*^ community (sampling zeros, *ω*_*ir*_ = 1) or not (structural zeros, *ω*_*ir*_ = 0). The indicator variable *ω*_*ir*_ was assumed a random Bernoulli variable with probability *Ω*_*r*_ that species *i* of the augmented array is a member of the *r*^*th*^ community of size *N*_*r*_ (i.e., species richness). The data augmentation technique converts the problem of estimating *N_r_* into the equivalent problem of estimating *Ω*_*r*_, and species richness is derived parameter by summing up the latent indicators ωir, since the expectation of *N*_*r*_ is equal to *M*_*r*_ *Ω*_*r*_. Inference at the community level was focused on modeled variation in *Ω*_*r*_ as a function of different covariates (see below). The observation model relates array ***Z*** to array ***Y***, such that *y*_*ijr*_ ~ Binomial (*K*_*jr*_, *p*_*ijr*_ *z*_*ijr*_), with *K_jr_* denoting the number of sampling occasions (i.e., camera-trap days for each site *j* in region *r*) and *p*_*ijr*_ the detection probability. Following this general model formulation, we investigated covariate effects at both community and species level.

Variations in region-specific species richness were investigated with a logit-link model that accounted for region-level variables. We assumed richness to vary in relation to human density (HDEN), distance to settlements (SETTL), available forest cover (FOREST) and forest patch size and compactness (P_SIZE) in the landscape. We also added the mean NDVI and the size of area sampled by camera-traps (AREA), as well as the coefficient of variation of annual precipitation (CV PREC) and a continent effect (CONTINENT, with Afrotropics as reference level) as proximate factors. Specifically, CONTINENT was treated as a fixed effect that accounted for the inherent community nestedness, while the use of local-scale covariates (i.e., area-specific variables) allowed us to capture contextual differences among regions in the same continent. Additionally, despite the overall large pairwise distances between regions (S5 Fig), we checked for potential spatial autocorrelation in species richness by performing a Moran’s I test [96] on both the entire data set and the subset of regions in the Afrotropics, where 2 pairs of regions were <100 km apart. We therefore found a lack of spatial autocorrelation both at the data set level (Moran’s I = 0.06; *p* = 0.23) and within the African continent (0.18; *p* = 0.11).


$$logit({Ω}_{r})=\beta 0+\beta 1*{NEOTROPICS}_{r}+\beta 2*{INDO-MALAYAN}_{r}+\beta 3*{AREA}_{r}+\beta 4*{NDVI}_{r}+\beta 5*{CV\;PREC}_{r}+\beta 6*{HDEN}_{r}+\beta 7*{SETTL}_{r}+\beta 8*{FOREST}_{r}+\beta 9*{P\_SIZE}_{r}$$

Global patterns of variations in occupancy probability were investigated by using species- and region-specific logit-linear model with region-level covariates [97]. We expected occupancy of species *i* in region *r* to vary in relation to the human density in the area (HDEN), the available forest habitat (FOREST), forest patch size and compactness (P_SIZE), and the mean distance to settlements (SETTL):

$$logit(\psi_{ir})=\theta 0_{ir}+\theta 1*{HDEN}_{r}+\theta 2*{FOREST}_{r}+\theta 3*{P\_SIZE}_{r}+\theta 4*{SETTL}_{r}+\varepsilon_{\psi ,ir}$$

$$with\;\varepsilon_{\psi ,ir}∼Normal(0,\sigma_{\psi ,ir})$$

Detection and occupancy probability were regressed using a logit-link function. We expected detection probability (*p*_*ijr*_) of species *i* at site *j* in region *r*, to be potentially affected by the camera trigger speed (CAM_TYPE) and by the distance from each *j* to the closest settlement (SETTL):

$$logit(p_{ijr})=\alpha 0_{ir}+\alpha 1*{CAM\_TYPE}_{jr}+\alpha 2*{SETTL}_{jr}+\varepsilon_{p,ir}$$

$$with\;\varepsilon_{p,ir}∼Normal(0,\sigma_{p,r})$$

Species- and region-specific variations in occupancy and detection probability were accommodated by random effects (ε_*ψ*,ir_ and ε_p,ir_) with region-specific random standard deviation (σ_*ψ*,r_ and σ_p,r_). This structure allowed us to improve the precision and the predictive capability of the model since data-deficient species can be accounted for by using information from data-rich species [94,98].

We fitted the multi-region community model in a Bayesian framework using Markov Chain Monte Carlo, and inference was based on 400,000 post-burn-in posterior samples (3 chains, default thinning of 1, and burn-in of 100,000). We assessed model effectiveness by verify chain convergence by using the R-hat diagnostic, with R-hat ≈ 1.01 for successful convergence. The hierarchical model was implemented in Nimble [99,100], through R [101]. For details on prior specification of model parameters, see the provided Nimble code.

### Ethics statement

Wildlife data used for this study are detections of mammals obtained non-invasively through camera-trapping. The study used data from the TEAM Network, eMAMMAL, and other research projects approved by the respective national authorities. Data collection in Tanzania was conducted under COSTECH and TAWIRI permission to F.R. and E.H.M. Research permits in Sarawak were given by Sarawak Forestry Corporation and Forest Department Sarawak (JM-A). Data collection in Myanmar was conducted in collaboration with the Ministry of Natural Resources and Environmental Conservation (MONREC) and with the Nature and Wildlife Conservation Division (NWCD). Data collection in Caxiuana was conducted in collaboration with the Museu Paraense Emílio Goeldi and Instituto Chico Mendes de Conservação da Biodiversidade (ICMBio). Data collection in Manas, India was conducted in collaboration with Directorate Manas Tiger Reserve, Department of Forests, Government of Assam. Data collection in RPPN Estação Veracel and Pau Brasil National Park (PBN) was authorized by SISBIO permit #60641. Data collection in Ecuador had the approval of Ecuador’s Ministry of Environment. Data collection in Uganda was conducted in collaboration with the Uganda Wildlife Authority (UWA), a body authorized to manage and research on wildlife in Uganda. Data collection in Gabon has been approved under the research authorization n°AR002/l 9/MESRS/CENAREST/CG/CST/SCAR. Data collection in Colombia was authorized by company owners, managers, or landowners. Data collection in Gabon was conducted under the research permits (AR0043/18; AE19004). Data collection in Sulawesi was permitted by the Ministry of Science and Technology (RISTEK) for permission to conduct the research in Indonesia.

## Supporting information

S1 Fig Graphical representation for Ranomafana National Park (Madagascar) of the 50 km buffer within which the variables of habitat loss, forest fragmentation, mean distance to infrastructure and human density have been calculated. The buffer expands from the camera-trap arrays [1,2] and is indicative of landscape-scale disturbance acting from both within and outside the protected areas. The green shape represents the national park border, while the black dots represent the camera-trap locations. Background map derived from OpenStreetMap (www.openstreetmap.org) through QGIS [3].
(DOCX)

S2 Fig Comparison between the percentage of forest cover (left chart) and average forest patch size (right) between the areas inside PA borders and outside the PA borders (i.e., the area within the 50 km buffer from the camera-trap arrays minus the PA extent). Charts show the values (black dots) only for the 31 study areas which are protected and the average ± SE. The differences are significant for both the percentage of available forest cover (Welch two-sample *t* test: *t* = 4.80, df = 56.86, *p*-value <0.001) and the average size of forest patches (*t* = 2.72, df = 37.53, *p*-value = 0.01). The data underlying this figure can be found in S4 Data.
(DOCX)

S3 Fig Map of the study areas, divided by those that form the TEAM Network (red dots) and those added for this study (yellow dots). The insets display examples of the sampling design for one TEAM (red) and non-TEAM area (yellow), both occurring in the Udzungwa Mountains of Tanzania. Tropical forest layer derived from Hansen and colleagues [1].
(DOCX)

S4 Fig Correlation matrix among the selected covariates (A) and variance inflation factor (VIF) scores (B). The data underlying this figure can be found in S5 Data.
(DOCX)

S5 Fig Distances (km) between pairs of areas for the Neotropics (above), Afrotropics (central), and Indo-Malayan tropics (below). The data underlying this figure can be found in S6 Data.
(DOCX)

S1 Table Summary of the main parameters of interest from the multi-region occupancy model. *α-*coefficients represent variables used to model the detection probability (*p*), *θ-*coefficients represent parameters used to model community occupancy probability (*ψ*), and the *β-*coefficients are parameters used to model species richness. Values are the mean of the posterior distribution, the related SD, and the 2.5%–97.5% Bayesian CI. Also included are the potential scale reduction statistics (R-hat), with values close to 1 indicating convergence of chains, and the number of samplings from the posterior distribution (n.eff).
(DOCX)

S2 Table List of the 11 areas for which there is evidence of recent local extinctions of mammals, along with values of forest cover and human density in the landscape and, in parentheses, their difference to the mean values for all 37 areas in the dataset expressed in %. Full details of target areas are in Table S4.
(DOCX)

S3 Table List of wild mammal species detected by camera-traps in the 37 study areas of the data set and included in the analyses. Species are listed in alphabetic order by taxonomic order.
(DOCX)

S4 Table List of the 37 areas included in the data set with location, management type, and environmental characteristics. Mean elevation (m.a.s.l.) of camera trap sites was sourced from the Global Biodiversity Information facility via package “rgbif” [1], mean annual precipitation (mm), and mean maximum temperature (°C) were sourced from the Worldclim historical monthly weather data [2], while the dominant landcover type was sourced from the MODIS Land Cover Type Yearly L3 Global 500 m. [3].
(DOCX)

S5 Table Details on sampling effort and design for the 37 areas included in the data set. “Sampling effort (camera days)” is calculated as the total number of 24-h periods camera traps (CT) worked; “Season” attributes if data collection was conducted during the dry (period of reduced precipitation) or the wet season (period where most of the yearly rain is concentrated).
(DOCX)

S1 Code R codes to reproduce the main manuscript’s figures. (TXT)

S2 Code Nimble code and model specifications for the multi-region community model. (TXT)

S1 Data Raw data underlying Fig 1, representing the observed and estimated specie richness for each sampled area. (XLSX)

S2 Data Raw data underlying Fig 3, representing the standardized beta coefficients for the effects of the predictors on species richness (A), and estimated predicted species richness in relation to the continent (B) and increasing human density (C). (XLSX)

S3 Data Raw data underlying Fig 4, representing the standardized beta coefficients for the effects of predictors on community occupancy (A), and estimated predicted community occupancy in relation to the increasing forest patch size (B) and increasing forest cover (C). (XLSX)

S4 Data Raw data underlying S2 Fig, representing the percentage of forest cover and average forest patch size, between the areas inside PA borders and outside the PA borders. (XLSX)

S5 Data Raw data underlying S4 Fig, representing the value of the correlation matrix among the selected covariates. (XLSX)

S6 Data Raw data underlying S5 Fig, representing the distances (km) between pairs of areas for the Neotropics, Afrotropics, and Indo-Malayan tropics. (XLSX)

## Abbreviations

BBS: Bukit Barisan
BCI: Barro Colorado
BIF: Bwind
CAX: Caxiuanã
CI: credible interval
COU: Cocha Cashu
CSN: Central Suriname
CV: coefficient of variation
DFN: Somalomo
DFS: Djoum
DVM: Danum Valley
GUR: Gurup
H: Hypothesis
HKK: Huai Kha Khaeng
INV: Ivindo
JAM: Jamar
KER: Kerinci Seblat
KRP: Korup
LEU: Gunung Leuser
LLA: Llanos
MAS: Manaus
MIN: Minziro
MNP: Manas
NAK: Nam Kading
NDVI: normalized difference vegetation index
NNN: Nouabalé Ndoki
PA: protected area
PBN: Pau Brasil/Veracel
P: Prediction
PSH: Pasoh
PWG: PWG-CEB logging concession
RKM: Rakhine
RNF: Ranomafana
SHS: Sagaing Htamanthi
SUL: Sulawes
TDM: Terra do Meio
TEAM: Tropical Ecology Assessment and Monitoring
TPN: Ta Phraya
UDZ: Udzungwa
UZS: Uzungwa scarp
VBA: Volcan Barva
VIF: variance inflation factor
VIR: Virunga
YAN: Yanachaga
YAS: Yasuni
