# Supplementary material for: Landscape-level human disturbance results in loss and contraction of mammalian populations in tropical forests
Source: PLoS Biol. 2025 Feb 13;23(2):e3002976. doi: 10.1371/journal.pbio.3002976 (PMC11825024; doi:10.1371/journal.pbio.3002976)
Supplement: S1 Fig — The buffer expands from the camera-trap arrays [1,2] and is indicative of landscape-scale disturbance acting from both within and outside the protected areas. The green shape represents the national park border, while the black dots represent the camera-trap locations. Background map derived from OpenStreetMap (www.openstreetmap.org) through QGIS [3]. (DOCX) [file pbio.3002976.s001.docx]

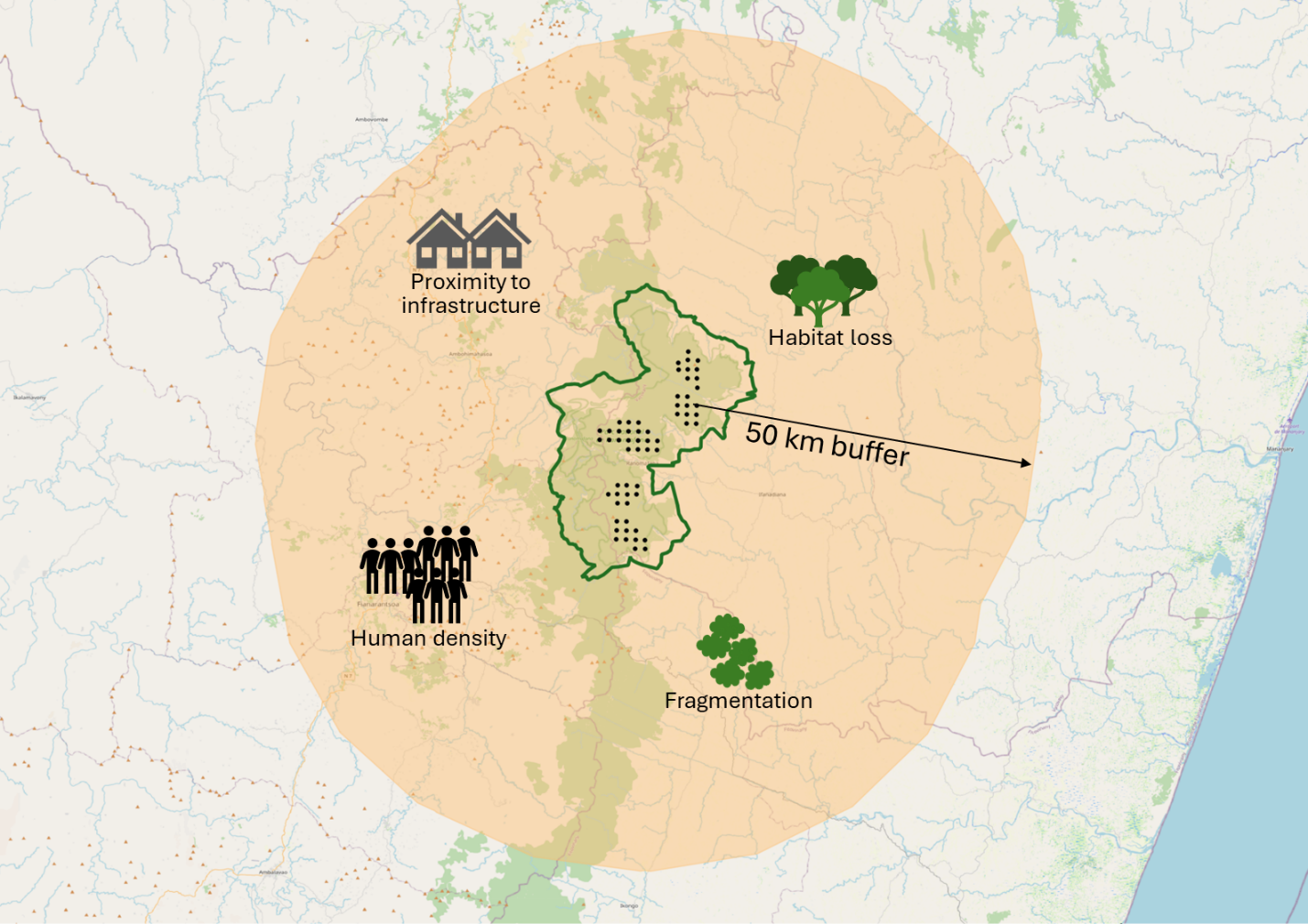


**S1 Fig** Graphical representation for Ranomafana National Park (Madagascar) of the 50km buffer within which the variables of habitat loss, forest fragmentation, mean distance to infrastructure and human density have been calculated. The buffer expands from the camera trap arrays [1,2] and is indicative of landscape-scale disturbance acting from both within and outside the protected areas. The green shape represents the national park border, while the black dots represent the camera trap locations. Background map derived from OpenStreetMap ([www.openstreetmap.org](http://www.openstreetmap.org)) through QGIS [3].

References

1. Semper-Pascual A, Sheil D, Beaudrot L, Dupont P, Dey S, Ahumada J, et al. Occurrence dynamics of mammals in protected tropical forests respond to human presence and activities. Nat Ecol Evol. 2023;7: 1092–1103. doi:10.1038/s41559-023-02060-6

2. Semper-Pascual A, Bischof R, Milleret C, Beaudrot L, Vallejo-Vargas AF, Ahumada JA, et al. Occupancy winners in tropical protected forests: a pantropical analysis. Proceedings of the Royal Society B: Biological Sciences. 2022;289: 20220457. doi:10.1098/rspb.2022.0457

3. QGIS Development Team. QGIS Geographic Information System. Open-source Geospatial 475 Foundation Project; 2019.
