# Supplementary material for: Landscape-level human disturbance results in loss and contraction of mammalian populations in tropical forests
Source: PLoS Biol. 2025 Feb 13;23(2):e3002976. doi: 10.1371/journal.pbio.3002976 (PMC11825024; doi:10.1371/journal.pbio.3002976)
Supplement: S2 Fig — Charts show the values (black dots) only for the 31 study areas which are protected and the average ± SE. The differences are significant for both the percentage of available forest cover (Welch two-sample t test: t = 4.80, df = 56.86, p-value <0.001) and the average size of forest patches (t = 2.72, df = 37.53, p-value = 0.01). The data underlying this figure can be found in S4 Data. (DOCX) [file pbio.3002976.s002.docx]

**
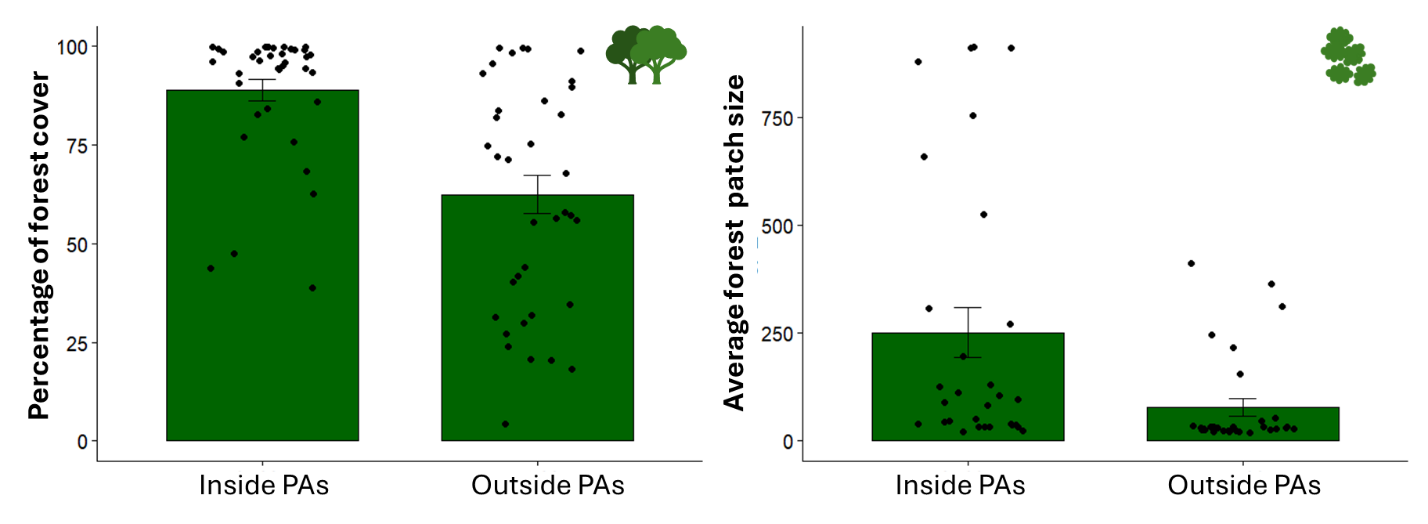
**

**S2 Fig** Comparison between the percentage of forest cover (left chart) and average forest patch size (right) between the areas inside PA borders and outside the PA borders (i.e., the area within the 50 km buffer from the camera trap arrays minus the PA extent). Charts show the values (black dots) only for the 31 study areas which are protected, and the average ± SE. The differences are significant for both the percentage of available forest cover (Welch Two Sample t-test: t = 4.80, df = 56.86, p-value < 0.001) and the average size of forest patches (t = 2.72, df = 37.53, p-value = 0.01). The data underlying this Figure can be found in S4 Data.
