# Supplementary material for: Landscape-level human disturbance results in loss and contraction of mammalian populations in tropical forests
Source: PLoS Biol. 2025 Feb 13;23(2):e3002976. doi: 10.1371/journal.pbio.3002976 (PMC11825024; doi:10.1371/journal.pbio.3002976)
Supplement: S3 Fig — The insets display examples of the sampling design for one TEAM (red) and non-TEAM area (yellow), both occurring in the Udzungwa Mountains of Tanzania. Tropical forest layer derived from Hansen and colleagues [1]. (DOCX) [file pbio.3002976.s003.docx]

S3
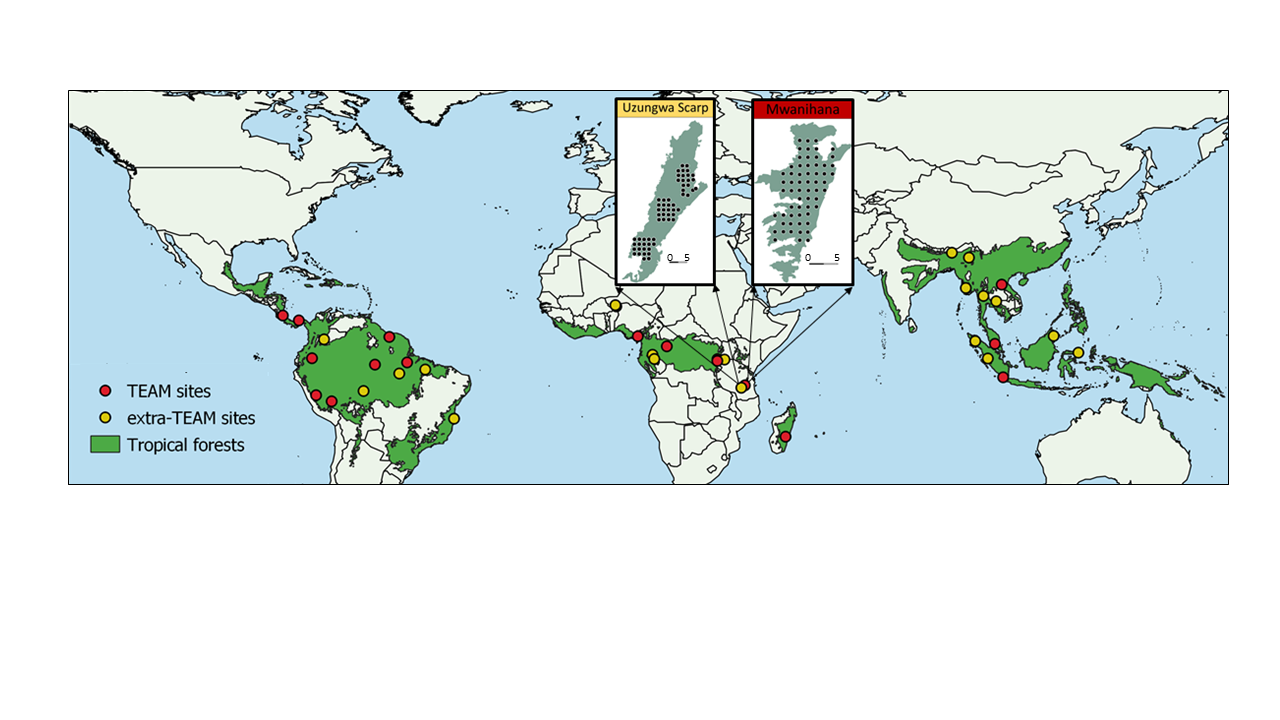
 Fig Map of the study areas, divided by those that form the TEAM Network (red dots) and those added for this study (yellow dots). The insets display examples of the sampling design for one TEAM (red) and non-TEAM area (yellow), both occurring in the Udzungwa Mountains of Tanzania. Tropical forest layer derived from Hansen et al., 2008 [1].

References

1. Hansen MC, Stehman SV, Potapov PV, Loveland TR, Townshend JRG, DeFries RS, et al. Humid tropical forest clearing from 2000 to 2005 quantified by using multitemporal and multiresolution remotely sensed data. Proceedings of the National Academy of Sciences. 2008;105: 9439–9444. doi:10.1073/pnas.0804042105
