# Supplementary material for: Landscape-level human disturbance results in loss and contraction of mammalian populations in tropical forests
Source: PLoS Biol. 2025 Feb 13;23(2):e3002976. doi: 10.1371/journal.pbio.3002976 (PMC11825024; doi:10.1371/journal.pbio.3002976)
Supplement: S5 Fig — The data underlying this figure can be found in S6 Data. (DOCX) [file pbio.3002976.s005.docx]

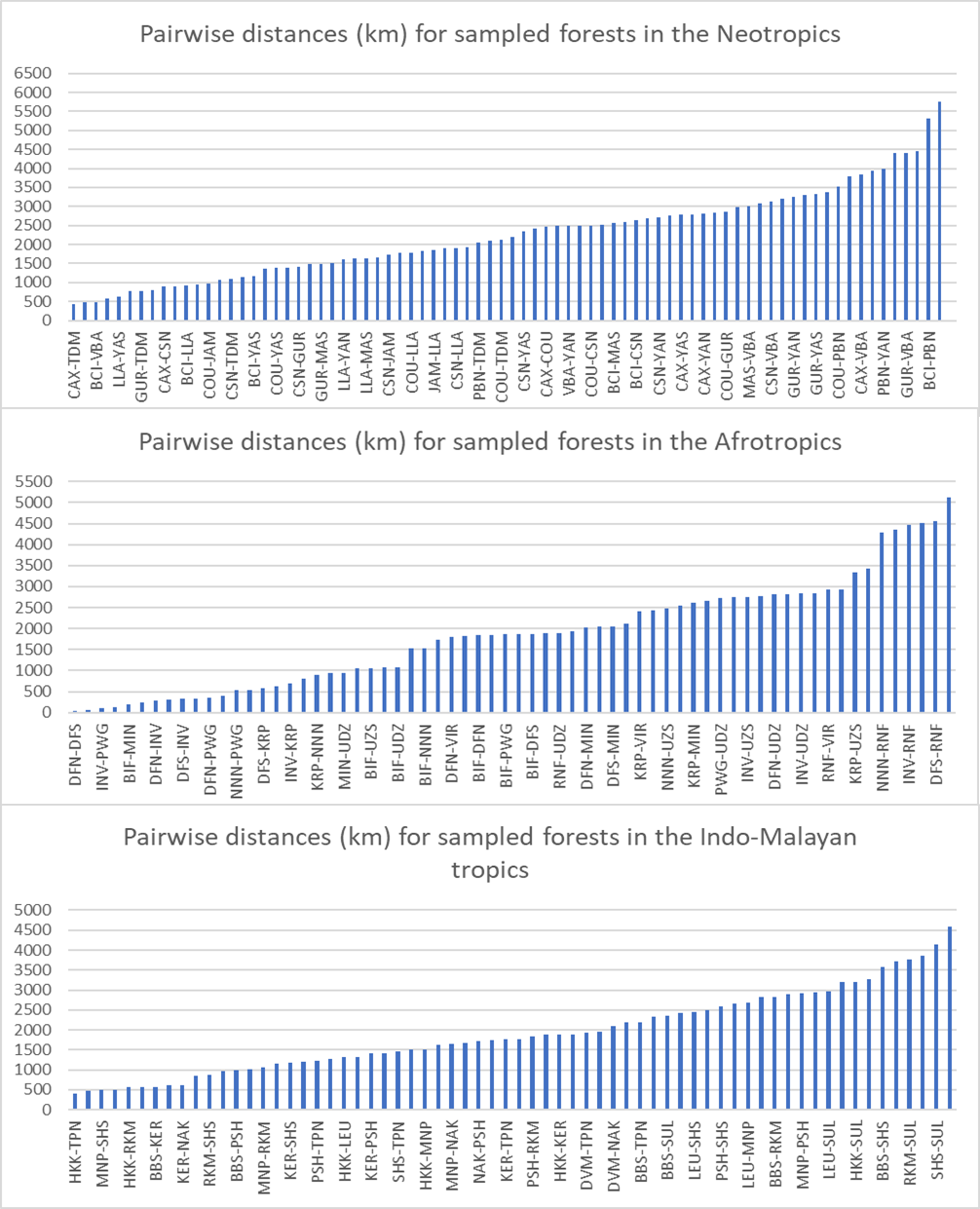


**S5 Fig** Distances (km) between pairs of areas for the Neotropics (above), Afrotropics (central) and Indo-Malayan tropics (below). The data underlying this Figure can be found in S6 Data.
