# Supplementary material for: Landscape-level human disturbance results in loss and contraction of mammalian populations in tropical forests
Source: PLoS Biol. 2025 Feb 13;23(2):e3002976. doi: 10.1371/journal.pbio.3002976 (PMC11825024; doi:10.1371/journal.pbio.3002976)
Supplement: S1 Table — α-coefficients represent variables used to model the detection probability (p), θ-coefficients represent parameters used to model community occupancy probability (ψ), and the β-coefficients are parameters used to model species richness. Values are the mean of the posterior distribution, the related SD, and the 2.5%–97.5% Bayesian CI. Also included are the potential scale reduction statistics (R-hat), with values close to 1 indicating convergence of chains, and the number of samplings from the posterior distribution (n.eff). (DOCX) [file pbio.3002976.s006.docx]

S1 Table

Summary of the main parameters of interest from the multi-region occupancy model. *α-*coefficients represent variables used to model the detection probability (*p*), *θ-*coefficients represent parameters used to model community occupancy probability (*ψ*), and the *β-*coefficients are parameters used to model species richness. Values are the mean of the posterior distribution, the related SD, and the 2.5% - 97.5% Bayesian CI. Also included are the potential scale reduction statistics (R-hat), with values close to 1 indicating convergence of chains, and the number of samplings from the posterior distribution (n.eff).

| **Submodel** | **Parameter** | **Variable** | **mean** | **sd** | **5%** | **50%** | **95%** | **R-hat** | **n.eff** |
| --- | --- | --- | --- | --- | --- | --- | --- | --- | --- |
| Detection | *α0* | Intercept | -3.97 | 0.10 | -4.15 | -3.96 | -3.84 | 1.02 | 308 |
|  | *α1* | Camera sensitivity | -0.16 | 0.08 | -0.29 | -0.16 | -0.06 | 1.00 | 1460 |
|  | *α2* | Proximity to infrastructures | -0.01 | 0.01 | -0.27 | -0.09 | 0.10 | 1.06 | 172 |
| Occupancy | ***θ****0* | Intercept | -0.93 | 0.10 | -1.10 | -0.93 | -0.81 | 1.01 | 609 |
|  | ***θ****1* | Human density | 0.01 | 0.13 | -0.21 | 0.01 | 0.17 | 1.01 | 531 |
|  | ***θ****2* | Forest patch size | 0.42 | 0.09 | 0.26 | 0.42 | 0.56 | 1.00 | 996 |
|  | ***θ****3* | Forest cover | 0.18 | 0.10 | 0.02 | 0.18 | 0.34 | 1.02 | 728 |
|  | ***θ****4* | Proximity to infrastructures | -0.06 | 0.13 | -0.27 | -0.05 | 0.11 | 1.00 | 572 |
| Richness | *β0* | Intercept | 0.04 | 0.14 | -0.19 | 0.04 | 0.28 | 1.00 | 8055 |
|  | *β1* | Neotropics | -0.29 | 0.18 | -0.60 | -0.29 | -0.01 | 1.00 | 10310 |
|  | *β2* | Indo-Malayan tropics | 0.06 | 0.21 | -0.29 | 0.06 | 0.40 | 1.00 | 9921 |
|  | *β3* | Sampled area | -0.06 | 0.07 | -0.18 | -0.06 | 0.06 | 1.00 | 12256 |
|  | *β4* | NDVI | -0.09 | 0.08 | -0.21 | -0.09 | 0.04 | 1.00 | 9173 |
|  | *β5* | CV precipitation | 0.01 | 0.07 | -0.10 | 0.01 | 0.13 | 1.00 | 11271 |
|  | *β6* | Human density | -0.15 | 0.08 | -0.28 | -0.15 | -0.02 | 1.00 | 6844 |
|  | *β7* | Proximity to infrastructures | 0.10 | 0.10 | -0.06 | 0.10 | 0.27 | 1.01 | 6641 |
|  | *β8* | Forest cover | 0.04 | 0.08 | -0.10 | 0.03 | 0.17 | 1.01 | 6788 |
|  | *β9* | Forest patch size | 0.08 | 0.08 | -0.04 | 0.08 | 0.21 | 1.01 | 7576 |
