# Supplementary material for: Landscape-level human disturbance results in loss and contraction of mammalian populations in tropical forests
Source: PLoS Biol. 2025 Feb 13;23(2):e3002976. doi: 10.1371/journal.pbio.3002976 (PMC11825024; doi:10.1371/journal.pbio.3002976)
Supplement: S2 Table — Full details of target areas are in Table S4. (DOCX) [file pbio.3002976.s007.docx]

**S2 Table**

List of the 11 areas for which there is evidence of recent local extinctions of mammals, along with values of forest cover and human density in the landscape and, in parentheses, their difference to the mean values for all 37 areas in the dataset expressed in %. Full details of target areas are in Table S4.

| **Biogeographic region** | **Area** | **Country** | **Species** | **Ref.** | **Forest cover ha** | **Human density persons/km^2^** |
| --- | --- | --- | --- | --- | --- | --- |
| Neotropics | Barro Colorado | Panama | White-lipped peccary (*Tayassu pecari*) Giant anteater (*Myrmecophaga tridactyla*) Jaguar (*Panthera onca*) | [1] | 454154 (-44%) | 279 (+258%) |
|  | Pau Brasil | Brazil | White-lipped peccary (*Tayassu pecari*) Giant armadillo (*Priodontes maximus*) Bush dog (*Speothos venaticu*s) Jaguar (*Panthera onca*) | M. Magioli, *pers. comm* | 359872 (-56%) | 39 (-51%) |
| Afrotropics | Bwindi | Uganda | Buffalo (*Syncerus caffer*) Leopard (Panthera pardus) Giant forest hog (*Hylochoerus meinertzhageni*) | [1] | 274653 (-66%) | 262 (+236%) |
|  | Minziro | Tanzania | Leopard (*Panthera pardus*) | [2] | 66841 (-92%) | 98 (+26%) |
|  | Uzungwa scarp | Tanzania | Leopard (*Panthera pardus*) Buffalo (*Syncerus caffer*) African elephant (*Loxodonta africana*) | [3] | 260166 (-68%) | 36 (-54%) |
| Indo-Malayan tropics | Bukit Barisan | Indonesia | Dhole (*Cuon alpinus*) Sumatran rhino (*Dicerorhinus sumatrensis*) | [4] | 366481 (-55%) | 149 (+90%) |
|  | Kerinci Seblat | Indonesia | Asian elephant (*Elephas maximus*) Sumatran rhino (*Dicerorhinus sumatrensis*) | [4] | 1173311 (+44%) | 65  (-17%) |
|  | Gunung Leuser | Indonesia | Dhole (*Cuon alpinus*) Sumatran rhino (*Dicerorhinus sumatrensis*) | [4] | 957266 (+18%) | 149 (+91%) |
|  | Nam Kading | Lao P.D.R. | Tiger (*Panthera tigris*) Leopard (*Panthera pardus*) | [1] | 766243 (-6%) | 31 (-60%) |
|  |  |  |  |  |  |  |
|  | Pasoh | Malaysia | Tiger (*Panthera tigris*) Asian elephant (*Elephas maximus*) Bearded pig (*Sus barbatus*) | [4] | 599768 (-26%) | 96 (+23%) |
|  | Sulawesi | Indonesia | Asian elephant (*Elephas maximus*) Sumatran rhino (*Dicerorhinus sumatrensis*) | C. Johnson *pers. comm* | 995310 (+22%) | 168 (+116%) |

References

1. Rovero F, Ahumada J, Jansen PA, Sheil D, Alvarez P, Boekee K, et al. A standardized assessment of forest mammal communities reveals consistent functional composition and vulnerability across the tropics. Ecography. 2020;43: 75–84. doi:10.1111/ecog.04773

2. Greco I, Rovero F. The African golden cat Caracal aurata in Tanzania: first record and vulnerability assessment. Oryx. 2021;55: 212–215. doi:10.1017/S003060532000040X

3. Oberosler V, Tenan S, Zipkin EF, Rovero F. Poor management in protected areas is associated with lowered tropical mammal diversity. Animal Conservation. 2020;23: 171–181. doi:10.1111/acv.12525

4. Amir Z, Moore JH, Negret PJ, Luskin MS. Megafauna extinctions produce idiosyncratic Anthropocene assemblages. Science Advances. 2022;8: eabq2307. doi:10.1126/sciadv.abq2307
