# Supplementary material for: Landscape-level human disturbance results in loss and contraction of mammalian populations in tropical forests
Source: PLoS Biol. 2025 Feb 13;23(2):e3002976. doi: 10.1371/journal.pbio.3002976 (PMC11825024; doi:10.1371/journal.pbio.3002976)
Supplement: S3 Table — Species are listed in alphabetic order by taxonomic order. (DOCX) [file pbio.3002976.s008.docx]

**S3 Table**

List of wild mammal species detected by camera-traps in the 37 study areas of the dataset and included in the analyses. Species are listed in alphabetic order by taxonomic order.

|  | **BIOGEOGRAPHIC REGIONS** | | | | | | | | | | | | | | | | | | | | | | | | | | | | | | | | | | | | |
| --- | --- | --- | --- | --- | --- | --- | --- | --- | --- | --- | --- | --- | --- | --- | --- | --- | --- | --- | --- | --- | --- | --- | --- | --- | --- | --- | --- | --- | --- | --- | --- | --- | --- | --- | --- | --- | --- |
|  | **Neotropics** | | | | | | | | | | | | | **Afrotropics** | | | | | | | | | | | | **Indo-Malayan tropics** | | | | | | | | | | | |
| **Species** | **GUR** | **JAM** | **TDM** | **PBN** | **LLA** | **BCI** | **YAS** | **CAX** | **CSN** | **YAN** | **COU** | **MAN** | **VBA** | **INV** | **UDZ** | **NNN** | **KRP** | **BIF** | **PWG** | **MIN** | **USZ** | **VIR** | **DFS** | **DFN** | **RNF** | **HKK** | **SHS** | **BBS** | **PSH** | **KER** | **LEU** | **RKM** | **DVM** | **TPH** | **SUL** | **MNP** | **NAK** |
| **N. observed species** | **21** | **19** | **25** | **18** | **18** | **19** | **26** | **21** | **24** | **23** | **26** | **20** | **13** | **25** | **26** | **29** | **22** | **23** | **29** | **22** | **20** | **12** | **34** | **27** | **14** | **32** | **23** | **21** | **24** | **30** | **26** | **14** | **26** | **19** | **7** | **22** | **15** |
| AFROSORCIDA |  |  |  |  |  |  |  |  |  |  |  |  |  |  |  |  |  |  |  |  |  |  |  |  |  |  |  |  |  |  |  |  |  |  |  |  |  |
| *Hemicentetes semispinosus* |  |  |  |  |  |  |  |  |  |  |  |  |  |  |  |  |  |  |  |  |  |  |  |  | RNF |  |  |  |  |  |  |  |  |  |  |  |  |
| *Tenrec ecaudatus* |  |  |  |  |  |  |  |  |  |  |  |  |  |  |  |  |  |  |  |  |  |  |  |  | RNF |  |  |  |  |  |  |  |  |  |  |  |  |
| ARTIODACTYLA |  |  |  |  |  |  |  |  |  |  |  |  |  |  |  |  |  |  |  |  |  |  |  |  |  |  |  |  |  |  |  |  |  |  |  |  |  |
| *Axis porcinus* |  |  |  |  |  |  |  |  |  |  |  |  |  |  |  |  |  |  |  |  |  |  |  |  |  |  |  |  |  |  |  |  |  |  |  | MNP |  |
| *Babyrousa celebensis* |  |  |  |  |  |  |  |  |  |  |  |  |  |  |  |  |  |  |  |  |  |  |  |  |  |  |  |  |  |  |  |  |  |  | SUL |  |  |
| *Bos gaurus* |  |  |  |  |  |  |  |  |  |  |  |  |  |  |  |  |  |  |  |  |  |  |  |  |  | HKK |  |  |  |  |  |  |  | TPH |  | MNP |  |
| *Bos javanicus* |  |  |  |  | LLA |  |  |  |  |  |  |  |  |  |  |  |  |  |  |  |  |  |  |  |  | HKK |  |  |  | KER |  |  |  |  |  |  |  |
| *Bubalus arnee* |  |  |  |  | LLA |  |  |  |  |  |  |  |  |  |  |  |  |  |  |  |  |  |  |  |  | HKK |  |  |  | KER |  |  |  |  |  | MNP |  |
| *Bubalus depressicornis* |  |  |  |  |  |  |  |  |  |  |  |  |  |  |  |  |  |  |  |  |  |  |  |  |  |  |  |  |  |  |  |  |  |  | SUL |  |  |
| *Capricornis milneedwardsii* |  |  |  |  |  |  |  |  |  |  |  |  |  |  |  |  |  |  |  |  |  |  |  |  |  |  | SHS |  |  |  |  |  |  | TPH |  |  |  |
| *Capricornis sumatraensis* |  |  |  |  |  |  |  |  |  |  |  |  |  |  |  |  |  |  |  |  |  |  |  |  |  |  |  |  | PSH | KER | LEU |  |  |  |  |  |  |
| *Cephalophus callipygus* |  |  |  |  |  |  |  |  |  |  |  |  |  | INV |  | NNN |  |  | PWG |  |  |  | DFS | DFN |  |  |  |  |  |  |  |  |  |  |  |  |  |
| *Cephalophus dorsalis* |  |  |  |  |  |  |  |  |  |  |  |  |  |  |  | NNN |  |  | PWG |  |  |  | DFS | DFN |  |  |  |  |  |  |  |  |  |  |  |  |  |
| *Cephalophus harveyi* |  |  |  |  |  |  |  |  |  |  |  |  |  |  | UDZ |  |  |  |  |  | USZ |  |  |  |  |  |  |  |  |  |  |  |  |  |  |  |  |
| *Cephalophus leucogaster* |  |  |  |  |  |  |  |  |  |  |  |  |  | INV |  | NNN |  |  | PWG |  |  |  | DFS |  |  |  |  |  |  |  |  |  |  |  |  |  |  |
| *Cephalophus nigrifrons* |  |  |  |  |  |  |  |  |  |  |  |  |  | INV |  | NNN |  | BIF | PWG |  |  | VIR |  |  |  |  |  |  |  |  |  |  |  |  |  |  |  |
| *Cephalophus ogilbyi* |  |  |  |  |  |  |  |  |  |  |  |  |  | INV |  |  | KRP |  | PWG |  |  |  |  |  |  |  |  |  |  |  |  |  |  |  |  |  |  |
| *Cephalophus silvicultor* |  |  |  |  |  |  |  |  |  |  |  |  |  | INV |  | NNN | KRP | BIF | PWG |  |  |  | DFS | DFN |  |  |  |  |  |  |  |  |  |  |  |  |  |
| *Cephalophus weynsi* |  |  |  |  |  |  |  |  |  |  |  |  |  |  |  |  |  | BIF |  |  |  |  |  |  |  |  |  |  |  |  |  |  |  |  |  |  |  |
| *Cephalophus spadix* |  |  |  |  |  |  |  |  |  |  |  |  |  |  | UDZ |  |  |  |  |  | USZ |  |  |  |  |  |  |  |  |  |  |  |  |  |  |  |  |
| *Kobus ellipsiprymnus* |  |  |  |  |  |  |  |  |  |  |  |  |  |  |  |  |  |  |  | MIN |  |  |  |  |  |  |  |  |  |  |  |  |  |  |  |  |  |
| *Neotragus batesi* |  |  |  |  |  |  |  |  |  |  |  |  |  |  |  |  |  |  |  |  |  |  | DFS | DFN |  |  |  |  |  |  |  |  |  |  |  |  |  |
| *Nesotragus moschatus* |  |  |  |  |  |  |  |  |  |  |  |  |  |  | UDZ |  |  |  |  |  | USZ |  |  |  |  |  |  |  |  |  |  |  |  |  |  |  |  |
| *Philantomba monticola* |  |  |  |  |  |  |  |  |  |  |  |  |  | INV |  | NNN | KRP |  | PWG | MIN | USZ |  | DFS | DFN |  |  |  |  |  |  |  |  |  |  |  |  |  |
| *Redunca redunca* |  |  |  |  |  |  |  |  |  |  |  |  |  |  |  |  |  |  |  | MIN |  |  |  |  |  |  |  |  |  |  |  |  |  |  |  |  |  |
| *Syncerus caffer* |  |  |  |  |  |  |  |  |  |  |  |  |  |  | UDZ |  |  |  | PWG | MIN |  | VIR | DFS | DFN |  |  |  |  |  |  |  |  |  |  |  |  |  |
| *Tragelaphus eurycerus* |  |  |  |  |  |  |  |  |  |  |  |  |  |  |  |  |  |  |  |  |  |  | DFS | DFN |  |  |  |  |  |  |  |  |  |  |  |  |  |
| *Tragelaphus scriptus* |  |  |  |  |  |  |  |  |  |  |  |  |  |  | UDZ |  |  | BIF | PWG | MIN | USZ | VIR |  |  |  |  |  |  |  |  |  |  |  |  |  |  |  |
| *Tragelaphus spekii* |  |  |  |  |  |  |  |  |  |  |  |  |  |  |  | NNN |  |  |  |  |  |  | DFS | DFN |  |  |  |  |  |  |  |  |  |  |  |  |  |
| *Mazama americana* | GUR |  | TDM |  |  |  | YAS | CAX | CSN | YAN | COU | MAN | |  |  |  |  |  |  |  |  |  |  |  |  |  |  |  |  |  |  |  |  |  |  |  |  |
| *Mazama gouazoubira* |  | JAM |  | PBN |  |  |  |  |  |  |  |  |  |  |  |  |  |  |  |  |  |  |  |  |  |  |  |  |  |  |  |  |  |  |  |  |  |
| *Mazama nemorivaga* | GUR |  | TDM |  |  |  | YAS | CAX | CSN |  |  | MAN | |  |  |  |  |  |  |  |  |  |  |  |  |  |  |  |  |  |  |  |  |  |  |  |  |
| *Mazama temama* |  |  |  |  |  | BCI | YAS |  |  |  |  |  | VBA |  |  |  |  |  |  |  |  |  |  |  |  |  |  |  |  |  |  |  |  |  |  |  |  |
| *Muntiacus atherodes* |  |  |  |  |  |  |  |  |  |  |  |  |  |  |  |  |  |  |  |  |  |  |  |  |  |  |  |  |  |  |  |  | DVM |  |  |  |  |
| *Muntiacus montanus* |  |  |  |  |  |  |  |  |  |  |  |  |  |  |  |  |  |  |  |  |  |  |  |  |  |  |  | BBS |  | KER | LEU |  |  |  |  |  |  |
| *Muntiacus muntjak* |  |  |  |  |  |  |  |  |  |  |  |  |  |  |  |  |  |  |  |  |  |  |  |  |  | HKK | SHS | BBS | PSH |  |  | RKM | DVM |  |  | MNP | NAK |
| *Muntiacus vaginalis* |  |  |  |  |  |  |  |  |  |  |  |  |  |  |  |  |  |  |  |  |  |  |  |  |  | HKK |  |  |  |  |  |  |  | TPH |  |  |  |
| *Odocoileus hemionus* |  |  |  |  | LLA |  |  |  |  |  |  |  |  |  |  |  |  |  |  |  |  |  |  |  |  |  |  |  |  |  |  |  |  |  |  |  |  |
| *Odocoileus virginianus* |  |  |  |  |  | BCI |  |  |  |  |  |  | VBA |  |  |  |  |  |  |  |  |  |  |  |  |  |  |  |  |  |  |  |  |  |  |  |  |
| *Rusa unicolor* |  |  |  |  |  |  |  |  |  |  |  |  |  |  |  |  |  |  |  |  |  |  |  |  |  | HKK | SHS | BBS | PSH | KER | LEU | RKM | DVM |  |  | MNP |  |
| *Hylochoerus meinertzhageni* |  |  |  |  |  |  |  |  |  |  |  |  |  |  |  | NNN |  |  |  |  |  |  |  |  |  |  |  |  |  |  |  |  |  |  |  |  |  |
| *Potamochoerus larvatus* |  |  |  |  |  |  |  |  |  |  |  |  |  |  | UDZ |  |  | BIF |  | MIN | USZ |  |  |  | RNF |  |  |  |  |  |  |  |  |  |  |  |  |
| *Potamochoerus porcus* |  |  |  |  |  |  |  |  |  |  |  |  |  | INV |  | NNN | KRP |  | PWG |  |  |  | DFS | DFN |  |  |  |  |  |  |  |  |  |  |  |  |  |
| *Sus barbatus* |  |  |  |  |  |  |  |  |  |  |  |  |  |  |  |  |  |  |  |  |  |  |  |  |  |  |  |  |  | KER |  |  | DVM |  |  |  |  |
| *Sus celebensis* |  |  |  |  |  |  |  |  |  |  |  |  |  |  |  |  |  |  |  |  |  |  |  |  |  |  |  |  |  |  |  |  |  |  | SUL |  |  |
| *Sus scrofa* |  |  |  |  | LLA |  |  |  |  |  |  |  |  |  |  |  |  |  |  |  |  |  |  |  |  | HKK | SHS | BBS | PSH | KER | LEU | RKM |  | TPH |  | MNP | NAK |
| *Hyemoschus aquaticus* |  |  |  |  |  |  |  |  |  |  |  |  |  | INV |  |  | KRP |  | PWG |  |  |  | DFS | DFN |  |  |  |  |  |  |  |  |  |  |  |  |  |
| *Tragulus javanicus* |  |  |  |  |  |  |  |  |  |  |  |  |  |  |  |  |  |  |  |  |  |  |  |  |  |  |  |  |  |  |  |  |  |  |  |  | NAK |
| *Tragulus kanchil* |  |  |  |  |  |  |  |  |  |  |  |  |  |  |  |  |  |  |  |  |  |  |  |  |  |  |  | BBS | PSH |  |  |  | DVM |  |  |  | NAK |
| *Tragulus napu* |  |  |  |  |  |  |  |  |  |  |  |  |  |  |  |  |  |  |  |  |  |  |  |  |  |  |  | BBS | PSH | KER | LEU |  |  |  |  |  |  |
| *Pecari tajacu* | GUR | JAM | TDM | PBN |  | BCI | YAS | CAX | CSN | YAN | COU | MAN | VBA |  |  |  |  |  |  |  |  |  |  |  |  |  |  |  |  |  |  |  |  |  |  |  |  |
| *Tayassu pecari* | GUR | JAM | TDM |  |  |  | YAS | CAX | CSN |  |  | MAN | |  |  |  |  |  |  |  |  |  |  |  |  |  |  |  |  |  |  |  |  |  |  |  |  |
| CARNIVORA |  |  |  |  |  |  |  |  |  |  |  |  | |  |  |  |  |  |  |  |  |  |  |  |  |  |  |  |  |  |  |  |  |  |  |  |  |
| *Atelocynus microtis* |  | JAM |  |  |  |  | YAS |  |  | YAN |  |  |  |  |  |  |  |  |  |  |  |  |  |  |  |  |  |  |  |  |  |  |  |  |  |  |  |
| *Canis adustus* |  |  |  |  |  |  |  |  |  |  |  |  |  |  |  |  |  | BIF |  |  |  | VIR |  |  |  |  |  |  |  |  |  |  |  |  |  |  |  |
| *Canis aureus* |  |  |  |  |  |  |  |  |  |  |  |  |  |  |  |  |  |  |  |  |  |  |  |  |  | HKK |  |  |  |  |  |  |  |  |  |  |  |
| *Canis latrans* |  |  |  |  |  | BCI |  |  |  |  |  |  |  |  |  |  |  |  |  |  |  |  |  |  |  |  |  |  |  |  |  |  |  |  |  |  |  |
| *Cerdocyon thous* |  | JAM | TDM | PBN | LLA |  |  |  |  |  |  |  |  |  |  |  |  |  |  |  |  |  |  |  |  |  |  |  |  |  |  |  |  |  |  |  |  |
| *Cuon alpinus* |  |  |  |  |  |  |  |  |  |  |  |  |  |  |  |  |  |  |  |  |  |  |  |  |  | HKK | SHS |  |  | KER |  | RKM |  | TPH |  | MNP |  |
| *Speothos venaticus* |  |  | TDM |  |  |  | YAS |  |  | YAN |  |  |  |  |  |  |  |  |  |  |  |  |  |  |  |  |  |  |  |  |  |  |  |  |  |  |  |
| *Cryptoprocta ferox* |  |  |  |  |  |  |  |  |  |  |  |  |  |  |  |  |  |  |  |  |  |  |  |  | RNF |  |  |  |  |  |  |  |  |  |  |  |  |
| *Fossa fossana* |  |  |  |  |  |  |  |  |  |  |  |  |  |  |  |  |  |  |  |  |  |  |  |  | RNF |  |  |  |  |  |  |  |  |  |  |  |  |
| *Galidia elegans* |  |  |  |  |  |  |  |  |  |  |  |  |  |  |  |  |  |  |  |  |  |  |  |  | RNF |  |  |  |  |  |  |  |  |  |  |  |  |
| *Galidictis fasciata* |  |  |  |  |  |  |  |  |  |  |  |  |  |  |  |  |  |  |  |  |  |  |  |  | RNF |  |  |  |  |  |  |  |  |  |  |  |  |
| *Atilax paludinosus* |  |  |  |  |  |  |  |  |  |  |  |  |  | INV | UDZ | NNN | KRP | BIF | PWG | MIN |  |  | DFS | DFN |  |  |  |  |  |  | LEU |  |  |  |  |  |  |
| *Bdeogale crassicauda* |  |  |  |  |  |  |  |  |  |  |  |  |  |  | UDZ |  |  |  |  |  | USZ |  |  |  |  |  |  |  |  |  |  |  |  |  |  |  |  |
| *Bdeogale nigripes* |  |  |  |  |  |  |  |  |  |  |  |  |  | INV |  | NNN | KRP |  | PWG |  |  |  | DFS | DFN |  |  |  |  |  |  |  |  |  |  |  |  |  |
| *Crossarchus obscurus* |  |  |  |  |  |  |  |  |  |  |  |  |  |  |  |  | KRP |  |  |  |  |  |  |  |  |  |  |  |  |  |  |  |  |  |  |  |  |
| *Crossarchus platycephalus* |  |  |  |  |  |  |  |  |  |  |  |  |  |  |  |  |  |  |  |  |  |  | DFS | DFN |  |  |  |  |  |  |  |  |  |  |  |  |  |
| *Caracal aurata* |  |  |  |  |  |  |  |  |  |  |  |  |  | INV |  | NNN |  | BIF | PWG | MIN |  | VIR | DFS |  |  |  |  |  |  |  |  |  |  |  |  |  |  |
| *Felis chaus* |  |  |  |  |  |  |  |  |  |  |  |  |  |  |  |  |  |  |  |  |  |  |  |  |  |  |  |  |  |  |  |  |  |  |  | MNP |  |
| *Leopardus guttulus* |  |  |  | PBN |  |  |  |  |  |  |  |  |  |  |  |  |  |  |  |  |  |  |  |  |  |  |  |  |  |  |  |  |  |  |  |  |  |
| *Leopardus pardalis* | GUR | JAM | TDM |  | LLA | BCI | YAS | CAX | CSN | YAN | COU | MAN | VBA |  |  |  |  |  |  |  |  |  |  |  |  |  |  |  |  |  |  |  |  |  |  |  |  |
| *Leopardus tigrinus* |  |  |  |  |  |  |  |  | CSN |  |  |  |  |  |  |  |  |  |  |  |  |  |  |  |  |  |  |  |  |  |  |  |  |  |  |  |  |
| *Leopardus wiedii* |  |  | TDM | PBN |  |  | YAS | CAX | CSN |  | COU |  |  |  |  |  |  |  |  |  |  |  |  |  |  |  |  |  |  |  |  |  |  |  |  |  |  |
| *Leptailurus serval* |  |  |  |  |  |  |  |  |  |  |  |  |  |  |  |  |  | BIF |  |  |  | VIR |  |  |  |  |  |  |  |  |  |  |  |  |  |  |  |
| *Neofelis nebulosa* |  |  |  |  |  |  |  |  |  |  |  |  |  |  |  |  |  |  |  |  |  |  |  |  |  |  | SHS |  |  | KER | LEU | RKM |  |  |  | MNP |  |
| *Neofelis diardi* |  |  |  |  |  |  |  |  |  |  |  |  |  |  |  |  |  |  |  |  |  |  |  |  |  |  |  | BBS |  |  |  |  |  |  |  |  |  |
| *Panthera onca* | GUR | JAM | TDM |  |  |  | YAS | CAX | CSN | YAN | COU | MAN | VBA |  |  |  |  |  |  |  |  |  |  |  |  |  |  |  |  |  |  |  |  |  |  |  |  |
| *Panthera pardus* |  |  |  |  |  |  |  |  |  |  |  |  |  | INV | UDZ | NNN |  |  | PWG |  |  |  | DFS |  |  | HKK |  |  | PSH |  |  |  |  |  |  | MNP |  |
| *Panthera tigris* |  |  |  |  |  |  |  |  |  |  |  |  |  |  |  |  |  |  |  |  |  |  |  |  |  | HKK | SHS | BBS |  | KER | LEU |  |  |  |  | MNP |  |
| *Pardofelis marmorata* |  |  |  |  |  |  |  |  |  |  |  |  |  |  |  |  |  |  |  |  |  |  |  |  |  |  | SHS | BBS |  | KER | LEU | RKM |  |  |  |  |  |
| *Pardofelis temminckii* |  |  |  |  |  |  |  |  |  |  |  |  |  |  |  |  |  |  |  |  |  |  |  |  |  | HKK |  | BBS |  | KER | LEU |  |  |  |  |  |  |
| *Prionailurus bengalensis* |  |  |  |  |  |  |  |  |  |  |  |  |  |  |  |  |  |  |  |  |  |  |  |  |  | HKK | SHS | BBS | PSH | KER | LEU | RKM | DVM | TPH |  | MNP | NAK |
| *Puma concolor* | GUR | JAM | TDM | PBN |  |  | YAS | CAX | CSN | YAN | COU | MAN | VBA |  |  |  |  |  |  |  |  |  |  |  |  |  |  |  |  |  |  |  |  |  |  |  |  |
| *Puma yagouaroundi* | GUR |  | TDM | PBN | LLA | BCI | YAS |  | CSN | YAN | COU |  |  |  |  |  |  |  |  |  |  |  |  |  |  |  |  |  |  |  |  |  |  |  |  |  |  |
| *Herpestes brachyurus* |  |  |  |  |  |  |  |  |  |  |  |  |  |  |  |  |  |  |  |  |  |  |  |  |  |  |  |  |  | KER | LEU |  | DVM |  |  |  |  |
| *Herpestes naso* |  |  |  |  |  |  |  |  |  |  |  |  |  |  |  |  | KRP |  | PWG |  |  |  | DFS | DFN |  |  |  |  |  |  |  |  |  |  |  |  |  |
| *Herpestes sanguineus* |  |  |  |  |  |  |  |  |  |  |  |  |  |  |  |  |  |  |  | MIN |  |  |  |  |  |  |  |  |  |  |  |  |  |  |  |  |  |
| *Herpestes urva* |  |  |  |  |  |  |  |  |  |  |  |  |  |  |  |  |  |  |  |  |  |  |  |  |  | HKK |  |  |  |  |  | RKM |  |  |  | MNP | NAK |
| *Herpestes javanicus* |  |  |  |  |  |  |  |  |  |  |  |  |  |  |  |  |  |  |  |  |  |  |  |  |  |  |  |  |  |  |  |  |  | TPH |  |  |  |
| *Mungos mungo* |  |  |  |  |  |  |  |  |  |  |  |  |  |  | UDZ |  |  |  |  |  | USZ |  |  |  |  |  |  |  |  |  |  |  |  |  |  |  |  |
| *Arctonyx collaris* |  |  |  |  |  |  |  |  |  |  |  |  |  |  |  |  |  |  |  |  |  |  |  |  |  | HKK |  |  |  | KER |  |  |  |  |  |  |  |
| *Eira barbara* | GUR | JAM | TDM | PBN |  | BCI | YAS | CAX | CSN | YAN | COU | MAN | VBA |  |  |  |  |  |  |  |  |  |  |  |  |  |  |  |  |  |  |  |  |  |  |  |  |
| *Galictis vittata* |  |  | TDM |  |  |  |  |  |  |  |  |  |  |  |  |  |  |  |  |  |  |  |  |  |  |  |  |  |  |  |  |  |  |  |  |  |  |
| *Martes flavigula* |  |  |  |  |  |  |  |  |  |  |  |  |  |  |  |  |  |  |  |  |  |  |  |  |  | HKK | SHS |  |  | KER | LEU |  |  | TPH |  | MNP |  |
| *Mellivora capensis* |  |  |  |  |  |  |  |  |  |  |  |  |  | INV | UDZ | NNN |  | BIF |  | MIN | USZ |  |  |  |  |  |  |  |  |  |  |  |  |  |  |  |  |
| *Melogale personata* |  |  |  |  |  |  |  |  |  |  |  |  |  |  |  |  |  |  |  |  |  |  |  |  |  | HKK |  |  |  |  |  |  |  | TPH |  |  |  |
| *Mustela strigidorsa* |  |  |  |  |  |  |  |  |  |  |  |  |  |  |  |  |  |  |  |  |  |  |  |  |  |  | SHS |  |  |  |  |  |  |  |  |  |  |
| *Mydaus javanensis* |  |  |  |  |  |  |  |  |  |  |  |  |  |  |  |  |  |  |  |  |  |  |  |  |  |  |  |  |  |  |  |  | DVM |  |  |  |  |
| *Nandinia binotata* |  |  |  |  |  |  |  |  |  |  |  |  |  | INV | UDZ | NNN | KRP | BIF | PWG | MIN | USZ |  | DFS | DFN |  |  |  |  |  |  |  |  |  |  |  |  |  |
| *Nasua narica* |  |  |  |  |  | BCI |  |  |  |  |  |  | VBA |  |  |  |  |  |  |  |  |  |  |  |  |  |  |  |  |  |  |  |  |  |  |  |  |
| *Nasua nasua* | GUR | JAM | TDM | PBN | LLA |  | YAS | CAX |  | YAN | COU | MAN | |  |  |  |  |  |  |  |  |  |  |  |  |  |  |  |  |  |  |  |  |  |  |  |  |
| *Potos flavus* |  |  |  | PBN |  |  |  |  |  |  |  |  |  |  |  |  |  |  |  |  |  |  |  |  |  |  |  |  |  |  |  |  |  |  |  |  |  |
| *Procyon cancrivorus* |  | JAM | TDM |  | LLA |  | YAS |  |  |  | COU |  |  |  |  |  |  |  |  |  |  |  |  |  |  |  |  |  |  |  |  |  |  |  |  |  |  |
| *Helarctos malayanus* |  |  |  |  |  |  |  |  |  |  |  |  |  |  |  |  |  |  |  |  |  |  |  |  |  | HKK | SHS | BBS | PSH | KER | LEU | RKM | DVM | TPH |  |  |  |
| *Ursus thibetanus* |  |  |  |  |  |  |  |  |  |  |  |  |  |  |  |  |  |  |  |  |  |  |  |  |  | HKK | SHS |  |  |  |  |  |  |  |  |  |  |
| *Arctictis binturong* |  |  |  |  |  |  |  |  |  |  |  |  |  |  |  |  |  |  |  |  |  |  |  |  |  |  |  |  |  | KER | LEU |  |  |  |  |  | NAK |
| *Civettictis civetta* |  |  |  |  |  |  |  |  |  |  |  |  |  | INV |  |  | KRP | BIF | PWG | MIN |  |  |  |  |  |  |  |  |  |  |  |  |  |  |  |  |  |
| *Cynogale bennettii* |  |  |  |  |  |  |  |  |  |  |  |  |  |  |  |  |  |  |  |  |  |  |  |  |  |  |  |  |  |  |  |  | DVM |  |  |  |  |
| *Genetta genetta* |  |  |  |  |  |  |  |  |  |  |  |  |  |  |  |  |  | BIF |  |  | USZ |  |  |  |  |  |  |  |  |  |  |  |  |  |  |  |  |
| *Genetta maculata* |  |  |  |  |  |  |  |  |  |  |  |  |  |  |  |  |  |  |  | MIN |  |  |  |  |  |  |  |  |  |  |  |  |  |  |  |  |  |
| *Genetta servalina* |  |  |  |  |  |  |  |  |  |  |  |  |  |  | UDZ | NNN | KRP | BIF | PWG |  | USZ | VIR | DFS | DFN |  |  |  |  |  |  |  |  |  |  |  |  |  |
| *Genetta tigrina* |  |  |  |  |  |  |  |  |  |  |  |  |  | INV |  |  |  |  |  |  |  |  | DFS |  |  |  |  |  |  |  |  |  |  |  |  |  |  |
| *Hemigalus derbyanus* |  |  |  |  |  |  |  |  |  |  |  |  |  |  |  |  |  |  |  |  |  |  |  |  |  |  |  | BBS | PSH | KER | LEU |  | DVM |  |  |  |  |
| *Macrogalidia musschenbroekii* |  |  |  |  |  |  |  |  |  |  |  |  |  |  |  |  |  |  |  |  |  |  |  |  |  |  |  |  |  |  |  |  |  |  | SUL |  |  |
| *Paguma larvata* |  |  |  |  |  |  |  |  |  |  |  |  |  |  |  |  |  |  |  |  |  |  |  |  |  | HKK |  | BBS |  | KER | LEU |  |  |  |  |  |  |
| *Paradoxurus hermaphroditus* |  |  |  |  |  |  |  |  |  |  |  |  |  |  |  |  |  |  |  |  |  |  |  |  |  | HKK | SHS | BBS |  | KER | LEU | RKM | DVM | TPH |  | MNP | NAK |
| *Viverra tangalunga* |  |  |  |  |  |  |  |  |  |  |  |  |  |  |  |  |  |  |  |  |  |  |  |  |  |  |  |  | PSH | KER |  |  | DVM |  | SUL |  |  |
| *Viverricula indica* |  |  |  |  |  |  |  |  |  |  |  |  |  |  |  |  |  |  |  |  |  |  |  |  |  |  |  |  |  |  |  |  |  | TPH |  | MNP |  |
| *Viverra zibetha* |  |  |  |  |  |  |  |  |  |  |  |  |  |  |  |  |  |  |  |  |  |  |  |  |  | HKK | SHS |  | PSH |  |  | RKM |  |  |  | MNP |  |
| CINGULATA |  |  |  |  |  |  |  |  |  |  |  |  |  |  |  |  |  |  |  |  |  |  |  |  |  |  |  |  |  |  |  |  |  |  |  |  |  |
| *Cabassous centralis* |  |  |  |  |  | BCI |  |  |  |  |  |  |  |  |  |  |  |  |  |  |  |  |  |  |  |  |  |  |  |  |  |  |  |  |  |  |  |
| *Cabassous tatouay* |  |  |  | PBN |  |  |  |  |  |  |  |  |  |  |  |  |  |  |  |  |  |  |  |  |  |  |  |  |  |  |  |  |  |  |  |  |  |
| *Cabassous unicinctus* | GUR |  |  |  | LLA |  |  |  |  | YAN |  |  |  |  |  |  |  |  |  |  |  |  |  |  |  |  |  |  |  |  |  |  |  |  |  |  |  |
| *Priodontes maximus* | GUR | JAM | TDM |  |  |  | YAS | CAX | CSN |  | COU |  |  |  |  |  |  |  |  |  |  |  |  |  |  |  |  |  |  |  |  |  |  |  |  |  |  |
| *Dasypus kappleri* |  |  |  |  |  |  | YAS | CAX | CSN | YAN | COU |  |  |  |  |  |  |  |  |  |  |  |  |  |  |  |  |  |  |  |  |  |  |  |  |  |  |
| *Dasypus novemcinctus* | GUR | JAM | TDM | PBN | LLA | BCI | YAS | CAX | CSN | YAN | COU | MAN | VBA |  |  |  |  |  |  |  |  |  |  |  |  |  |  |  |  |  |  |  |  |  |  |  |  |
| DIDELPHIMORPHIA |  |  |  |  |  |  |  |  |  |  |  |  |  |  |  |  |  |  |  |  |  |  |  |  |  |  |  |  |  |  |  |  |  |  |  |  |  |
| *Didelphis aurita* |  |  |  | PBN |  |  |  |  |  |  |  |  |  |  |  |  |  |  |  |  |  |  |  |  |  |  |  |  |  |  |  |  |  |  |  |  |  |
| *Didelphis marsupialis* | GUR | JAM | TDM |  | LLA | BCI | YAS | CAX | CSN | YAN | COU | MAN | VBA |  |  |  |  |  |  |  |  |  |  |  |  |  |  |  |  |  |  |  |  |  |  |  |  |
| *Metachirus nudicaudatus* | GUR |  |  |  |  | BCI |  | CAX | CSN |  |  | MAN | |  |  |  |  |  |  |  |  |  |  |  |  |  |  |  |  |  |  |  |  |  |  |  |  |
| *Philander mondolfii* |  |  |  |  |  |  |  |  |  |  |  |  |  |  |  | NNN |  |  |  |  |  |  |  |  |  |  |  |  |  |  |  |  |  |  |  |  |  |
| *Philander opossum* |  |  |  |  |  | BCI |  |  | CSN |  | COU | MAN | |  |  |  |  |  |  |  |  |  |  |  |  |  |  |  |  |  |  |  |  |  |  |  |  |
| EULIPOTYPHLA |  |  |  |  |  |  |  |  |  |  |  |  | |  |  |  |  |  |  |  |  |  |  |  |  |  |  |  |  |  |  |  |  |  |  |  |  |
| *Echinosorex gymnura* |  |  |  |  |  |  |  |  |  |  |  |  |  |  |  |  |  |  |  |  |  |  |  |  |  |  |  |  | PSH |  | LEU |  | DVM |  |  |  |  |
| HYRACOIDEA |  |  |  |  |  |  |  |  |  |  |  |  |  |  |  |  |  |  |  |  |  |  |  |  |  |  |  |  |  |  |  |  |  |  |  |  |  |
| *Dendrohyrax arboreus* |  |  |  |  |  |  |  |  |  |  |  |  |  |  |  |  |  |  |  |  |  | VIR |  |  |  |  |  |  |  |  |  |  |  |  |  |  |  |
| *Dendrohyrax validus* |  |  |  |  |  |  |  |  |  |  |  |  |  |  | UDZ |  |  |  |  |  | USZ |  |  |  |  |  |  |  |  |  |  |  |  |  |  |  |  |
| LAGOMORPHA |  |  |  |  |  |  |  |  |  |  |  |  |  |  |  |  |  |  |  |  |  |  |  |  |  |  |  |  |  |  |  |  |  |  |  |  |  |
| *Caprolagus hispidus* |  |  |  |  |  |  |  |  |  |  |  |  |  |  |  |  |  |  |  |  |  |  |  |  |  |  |  |  |  |  |  |  |  |  |  | MNP |  |
| *Lepus nigricollis* |  |  |  |  |  |  |  |  |  |  |  |  |  |  |  |  |  |  |  |  |  |  |  |  |  |  |  |  |  |  |  |  |  |  |  | MNP |  |
| *Sylvilagus brasiliensis* |  |  |  | PBN |  | BCI |  |  |  |  | COU |  |  |  |  |  |  |  |  |  |  |  |  |  |  |  |  |  |  |  |  |  |  |  |  |  |  |
| MACROSCELIDAE |  |  |  |  |  |  |  |  |  |  |  |  |  |  |  |  |  |  |  |  |  |  |  |  |  |  |  |  |  |  |  |  |  |  |  |  |  |
| *Petrodromus tetradactylus* |  |  |  |  |  |  |  |  |  |  |  |  |  |  | UDZ |  |  |  |  |  | USZ |  |  |  |  |  |  |  |  |  |  |  |  |  |  |  |  |
| *Rhynchocyon cirnei* |  |  |  |  |  |  |  |  |  |  |  |  |  |  | UDZ |  |  |  |  |  | USZ |  |  |  |  |  |  |  |  |  |  |  |  |  |  |  |  |
| *Rhynchocyon udzungwensis* |  |  |  |  |  |  |  |  |  |  |  |  |  |  | UDZ |  |  |  |  |  |  |  |  |  |  |  |  |  |  |  |  |  |  |  |  |  |  |
| PERISSODACTYA |  |  |  |  |  |  |  |  |  |  |  |  |  |  |  |  |  |  |  |  |  |  |  |  |  |  |  |  |  |  |  |  |  |  |  |  |  |
| *Rhinoceros unicornis* |  |  |  |  |  |  |  |  |  |  |  |  |  |  |  |  |  |  |  |  |  |  |  |  |  |  |  |  |  |  |  |  |  |  |  | MNP |  |
| *Rhinosciurus laticaudatus* |  |  |  |  |  |  |  |  |  |  |  |  |  |  |  |  |  |  |  |  |  |  |  |  |  |  |  |  | PSH |  |  |  |  |  |  |  |  |
| *Tapirus bairdii* |  |  |  |  |  |  |  |  |  |  |  |  | VBA |  |  |  |  |  |  |  |  |  |  |  |  |  |  |  |  |  |  |  |  |  |  |  |  |
| *Tapirus indicus* |  |  |  |  |  |  |  |  |  |  |  |  |  |  |  |  |  |  |  |  |  |  |  |  |  | HKK |  | BBS | PSH | KER |  |  |  |  |  |  |  |
| *Tapirus terrestris* | GUR | JAM | TDM | PBN |  |  | YAS | CAX | CSN | YAN | COU | MAN | |  |  |  |  |  |  |  |  |  |  |  |  |  |  |  |  |  |  |  |  |  |  |  |  |
| PHOLIDOTA |  |  |  |  |  |  |  |  |  |  |  |  | |  |  |  |  |  |  |  |  |  |  |  |  |  |  |  |  |  |  |  |  |  |  |  |  |
| *Manis javanica* |  |  |  |  |  |  |  |  |  |  |  |  |  |  |  |  |  |  |  |  |  |  |  |  |  | HKK | SHS |  | PSH |  | LEU |  | DVM | TPH |  |  |  |
| *Manis pentadactyla* |  |  |  |  |  |  |  |  |  |  |  |  |  |  |  |  |  |  |  |  |  |  |  |  |  |  | SHS |  |  |  |  |  |  |  |  |  |  |
| *Phataginus tricuspis* |  |  |  |  |  |  |  |  |  |  |  |  |  | INV |  | NNN | KRP | BIF | PWG | MIN |  |  | DFS | DFN |  |  |  |  |  |  |  |  |  |  |  |  |  |
| *Smutsia gigantea* |  |  |  |  |  |  |  |  |  |  |  |  |  | INV |  | NNN |  |  | PWG | MIN |  |  | DFS | DFN |  |  |  |  |  |  |  |  |  |  |  |  |  |
| PILOSA |  |  |  |  |  |  |  |  |  |  |  |  |  |  |  |  |  |  |  |  |  |  |  |  |  |  |  |  |  |  |  |  |  |  |  |  |  |
| *Myrmecophaga tridactyla* | GUR | JAM | TDM |  | LLA |  | YAS | CAX | CSN | YAN | COU | MAN | |  |  |  |  |  |  |  |  |  |  |  |  |  |  |  |  |  |  |  |  |  |  |  |  |
| *Tamandua mexicana* |  |  |  |  |  | BCI |  |  |  |  |  |  |  |  |  |  |  |  |  |  |  |  |  |  |  |  |  |  |  |  |  |  |  |  |  |  |  |
| *Tamandua tetradactyla* | GUR | JAM | TDM | PBN | LLA |  | YAS | CAX | CSN | YAN |  | MAN | |  |  |  |  |  |  |  |  |  |  |  |  |  |  |  |  |  |  |  |  |  |  |  |  |
| PRIMATES |  |  |  |  |  |  |  |  |  |  |  |  | |  |  |  |  |  |  |  |  |  |  |  |  |  |  |  |  |  |  |  |  |  |  |  |  |
| *Lagothrix cana* |  |  |  |  |  |  |  |  |  | YAN |  |  |  |  |  |  |  |  |  |  |  |  |  |  |  |  |  |  |  |  |  |  |  |  |  |  |  |
| *Saguinus fuscicollis* |  |  |  |  |  |  |  |  |  |  | COU |  |  |  |  |  |  |  |  |  |  |  |  |  |  |  |  |  |  |  |  |  |  |  |  |  |  |
| *Cercocebus agilis* |  |  |  |  |  |  |  |  |  |  |  |  |  |  |  | NNN |  |  |  |  |  |  | DFS | DFN |  |  |  |  |  |  |  |  |  |  |  |  |  |
| *Cercocebus sanjei* |  |  |  |  |  |  |  |  |  |  |  |  |  |  | UDZ |  |  |  |  |  | USZ |  |  |  |  |  |  |  |  |  |  |  |  |  |  |  |  |
| *Cercocebus torquatus* |  |  |  |  |  |  |  |  |  |  |  |  |  |  |  |  | KRP |  |  |  |  |  |  |  |  |  |  |  |  |  |  |  |  |  |  |  |  |
| *Cercopithecus ascanius* |  |  |  |  |  |  |  |  |  |  |  |  |  |  |  |  |  |  |  | MIN |  |  |  | DFN |  |  |  |  |  |  |  |  |  |  |  |  |  |
| *Cercopithecus cephus* |  |  |  |  |  |  |  |  |  |  |  |  |  |  |  | NNN |  |  |  |  |  |  |  | DFN |  |  |  |  |  |  |  |  |  |  |  |  |  |
| *Cercopithecus lhoesti* |  |  |  |  |  |  |  |  |  |  |  |  |  |  |  |  |  | BIF |  |  |  |  |  |  |  |  |  |  |  |  |  |  |  |  |  |  |  |
| *Cercopithecus mitis* |  |  |  |  |  |  |  |  |  |  |  |  |  |  | UDZ |  |  |  |  | MIN | USZ | VIR |  |  |  |  |  |  |  |  |  |  |  |  |  |  |  |
| *Cercopithecus nictitans* |  |  |  |  |  |  |  |  |  |  |  |  |  | INV |  | NNN |  |  |  |  |  |  |  |  |  |  |  |  |  |  |  |  |  |  |  |  |  |
| *Cercopithecus pogonias* |  |  |  |  |  |  |  |  |  |  |  |  |  | INV |  |  |  |  |  |  |  |  |  |  |  |  |  |  |  |  |  |  |  |  |  |  |  |
| *Lophocebus albigena* |  |  |  |  |  |  |  |  |  |  |  |  |  |  |  |  |  |  |  | MIN |  |  |  |  |  |  |  |  |  |  |  |  |  |  |  |  |  |
| *Macaca arctoides* |  |  |  |  |  |  |  |  |  |  |  |  |  |  |  |  |  |  |  |  |  |  |  |  |  | HKK | SHS |  |  |  |  |  |  |  |  |  | NAK |
| *Macaca fascicularis* |  |  |  |  |  |  |  |  |  |  |  |  |  |  |  |  |  |  |  |  |  |  |  |  |  | HKK |  |  | PSH | KER | LEU |  | DVM |  |  |  |  |
| *Macaca leonina* |  |  |  |  |  |  |  |  |  |  |  |  |  |  |  |  |  |  |  |  |  |  |  |  |  | HKK | SHS |  |  |  |  | RKM |  | TPH |  |  | NAK |
| *Macaca mulatta* |  |  |  |  |  |  |  |  |  |  |  |  |  |  |  |  |  |  |  |  |  |  |  |  |  |  | SHS |  |  |  |  |  |  |  |  |  |  |
| *Macaca nemestrina* |  |  |  |  |  |  |  |  |  |  |  |  |  |  |  |  |  |  |  |  |  |  |  |  |  |  |  | BBS | PSH | KER | LEU |  | DVM |  |  |  |  |
| *Macaca nigra* |  |  |  |  |  |  |  |  |  |  |  |  |  |  |  |  |  |  |  |  |  |  |  |  |  |  |  |  |  |  |  |  |  |  | SUL |  |  |
| *Macaca nigrescens* |  |  |  |  |  |  |  |  |  |  |  |  |  |  |  |  |  |  |  |  |  |  |  |  |  |  |  |  |  |  |  |  |  |  | SUL |  |  |
| *Mandrillus leucophaeus* |  |  |  |  |  |  |  |  |  |  |  |  |  |  |  |  | KRP |  |  |  |  |  | DFS |  |  |  |  |  |  |  |  |  |  |  |  |  |  |
| *Papio anubis* |  |  |  |  |  |  |  |  |  |  |  |  |  |  |  |  |  | BIF |  | MIN |  |  |  |  |  |  |  |  |  |  |  |  |  |  |  |  |  |
| *Papio cynocephalus* |  |  |  |  |  |  |  |  |  |  |  |  |  |  | UDZ |  |  |  |  |  | USZ |  |  |  |  |  |  |  |  |  |  |  |  |  |  |  |  |
| *Presbytis melalophos* |  |  |  |  |  |  |  |  |  |  |  |  |  |  |  |  |  |  |  |  |  |  |  |  |  |  |  | BBS |  | KER |  |  |  |  |  |  |  |
| *Procolobus gordonorum* |  |  |  |  |  |  |  |  |  |  |  |  |  |  | UDZ |  |  |  |  |  |  |  |  |  |  |  |  |  |  |  |  |  |  |  |  |  |  |
| *Trachypithecus obscurus* |  |  |  |  |  |  |  |  |  |  |  |  |  |  |  |  |  |  |  |  |  |  |  |  |  |  |  |  |  | KER | LEU |  |  |  |  |  |  |
| *Saimiri boliviensis* |  |  |  |  |  |  |  |  |  |  | COU |  |  |  |  |  |  |  |  |  |  |  |  |  |  |  |  |  |  |  |  |  |  |  |  |  |  |
| *Saimiri sciureus* |  |  | TDM |  | LLA |  |  |  |  |  |  |  |  |  |  |  |  |  |  |  |  |  |  |  |  |  |  |  |  |  |  |  |  |  |  |  |  |
| *Pan troglodytes* |  |  |  |  |  |  |  |  |  |  |  |  |  | INV |  | NNN |  | BIF | PWG |  |  |  | DFS | DFN |  |  |  |  |  |  |  |  |  |  |  |  |  |
| *Pongo abelii* |  |  |  |  |  |  |  |  |  |  |  |  |  |  |  |  |  |  |  |  |  |  |  |  |  |  |  |  |  |  | LEU |  | DVM |  |  |  |  |
| *Eulemur fulvus* |  |  |  |  |  |  |  |  |  |  |  |  |  |  |  |  |  |  |  |  |  |  |  |  | RNF |  |  |  |  |  |  |  |  |  |  |  |  |
| *Eulemur rufifrons* |  |  |  |  |  |  |  |  |  |  |  |  |  |  |  |  |  |  |  |  |  |  |  |  | RNF |  |  |  |  |  |  |  |  |  |  |  |  |
| *Gorilla beringei* |  |  |  |  |  |  |  |  |  |  |  |  |  |  |  |  |  | BIF |  |  |  | VIR |  |  |  |  |  |  |  |  |  |  |  |  |  |  |  |
| *Gorilla gorilla* |  |  |  |  |  |  |  |  |  |  |  |  |  | INV |  | NNN |  |  | PWG |  |  |  | DFS |  |  |  |  |  |  |  |  |  |  |  |  |  |  |
| *Hapalemur aureus* |  |  |  |  |  |  |  |  |  |  |  |  |  |  |  |  |  |  |  |  |  |  |  |  | RNF |  |  |  |  |  |  |  |  |  |  |  |  |
| *Hapalemur griseus* |  |  |  |  |  |  |  |  |  |  |  |  |  |  |  |  |  |  |  |  |  |  |  |  | RNF |  |  |  |  |  |  |  |  |  |  |  |  |
| PROBOSCIDEA |  |  |  |  |  |  |  |  |  |  |  |  |  |  |  |  |  |  |  |  |  |  |  |  |  |  |  |  |  |  |  |  |  |  |  |  |  |
| *Elephas maximus* |  |  |  |  |  |  |  |  |  |  |  |  |  |  |  |  |  |  |  |  |  |  |  |  |  | HKK | SHS | BBS |  |  | LEU | RKM | DVM |  |  | MNP |  |
| *Loxodonta africana* |  |  |  |  |  |  |  |  |  |  |  |  |  |  | UDZ | NNN | KRP | BIF |  | MIN |  | VIR | DFS |  |  |  |  |  |  |  |  |  |  |  |  |  |  |
| RODENTIA |  |  |  |  |  |  |  |  |  |  |  |  |  |  |  |  |  |  |  |  |  |  |  |  |  |  |  |  |  |  |  |  |  |  |  |  |  |
| *Galea spixii* | GUR |  |  |  |  |  |  |  |  |  |  |  |  |  |  |  |  |  |  |  |  |  |  |  |  |  |  |  |  |  |  |  |  |  |  |  |  |
| *Hydrochoerus hydrochaeris* |  |  |  |  | LLA |  |  |  |  |  |  |  |  |  |  |  |  |  |  |  |  |  |  |  |  |  |  |  |  |  |  |  |  |  |  |  |  |
| *Euryoryzomys nitidus* |  |  |  |  |  |  |  |  |  |  | COU |  |  |  |  |  |  |  |  |  |  |  |  |  |  |  |  |  |  |  |  |  |  |  |  |  |  |
| *Tylomys watsoni* |  |  |  |  |  |  |  |  |  | YAN |  |  |  |  |  |  |  |  |  |  |  |  |  |  |  |  |  |  |  |  |  |  |  |  |  |  |  |
| *Cuniculus paca* | GUR | JAM | TDM | PBN | LLA | BCI | YAS | CAX | CSN | YAN | COU | MAN | VBA |  |  |  |  |  |  |  |  |  |  |  |  |  |  |  |  |  |  |  |  |  |  |  |  |
| *Dasyprocta fuliginosa* |  |  |  |  | LLA |  | YAS |  |  | YAN |  |  |  |  |  |  |  |  |  |  |  |  |  |  |  |  |  |  |  |  |  |  |  |  |  |  |  |
| *Dasyprocta leporina* |  |  |  | PBN |  |  |  | CAX | CSN |  |  | MAN | |  |  |  |  |  |  |  |  |  |  |  |  |  |  |  |  |  |  |  |  |  |  |  |  |
| *Dasyprocta prymnolopha* | GUR |  |  |  |  |  |  |  |  |  |  |  |  |  |  |  |  |  |  |  |  |  |  |  |  |  |  |  |  |  |  |  |  |  |  |  |  |
| *Dasyprocta punctata* |  | JAM |  |  |  | BCI |  |  |  |  | COU |  | VBA |  |  |  |  |  |  |  |  |  |  |  |  |  |  |  |  |  |  |  |  |  |  |  |  |
| *Dasyprocta ruatanica* |  |  | TDM |  |  |  |  |  |  |  |  |  |  |  |  |  |  |  |  |  |  |  |  |  |  |  |  |  |  |  |  |  |  |  |  |  |  |
| *Myoprocta acouchy* |  |  |  |  |  |  |  |  | CSN |  |  | MAN | |  |  |  |  |  |  |  |  |  |  |  |  |  |  |  |  |  |  |  |  |  |  |  |  |
| *Myoprocta pratti* |  |  |  |  |  |  | YAS |  |  |  | COU |  |  |  |  |  |  |  |  |  |  |  |  |  |  |  |  |  |  |  |  |  |  |  |  |  |  |
| *Dactylomys peruanus* |  |  | TDM |  |  |  |  |  |  |  |  |  |  |  |  |  |  |  |  |  |  |  |  |  |  |  |  |  |  |  |  |  |  |  |  |  |  |
| *Proechimys brevicauda* |  |  |  |  |  |  |  |  |  |  | COU |  |  |  |  |  |  |  |  |  |  |  |  |  |  |  |  |  |  |  |  |  |  |  |  |  |  |
| *Proechimys semispinosus* |  |  |  |  |  | BCI |  | CAX |  |  |  |  |  |  |  |  |  |  |  |  |  |  |  |  |  |  |  |  |  |  |  |  |  |  |  |  |  |
| *Coendou prehensilis* |  |  |  |  |  |  |  |  |  | YAN |  |  |  |  |  |  |  |  |  |  |  |  |  |  |  |  |  |  |  |  |  |  |  |  |  |  |  |
| *Atherurus africanus* |  |  |  |  |  |  |  |  |  |  |  |  |  | INV |  | NNN | KRP | BIF | PWG |  |  |  | DFS | DFN |  |  |  |  |  |  |  |  |  |  |  |  |  |
| *Atherurus macrourus* |  |  |  |  |  |  |  |  |  |  |  |  |  |  |  |  |  |  |  |  |  |  |  |  |  | HKK | SHS |  | PSH |  |  |  |  | TPH |  |  | NAK |
| *Hystrix africaeaustralis* |  |  |  |  |  |  |  |  |  |  |  |  |  |  | UDZ |  |  |  |  |  |  |  |  |  |  |  |  |  |  |  |  |  |  |  |  |  |  |
| *Hystrix brachyura* |  |  |  |  |  |  |  |  |  |  |  |  |  |  |  |  |  |  |  |  |  |  |  |  |  | HKK | SHS | BBS | PSH | KER | LEU | RKM | DVM | TPH |  |  | NAK |
| *Hystrix crassispinis* |  |  |  |  |  |  |  |  |  |  |  |  |  |  |  |  |  |  |  |  |  |  |  |  |  |  |  |  |  |  |  |  | DVM |  |  |  |  |
| *Hystrix indica* |  |  |  |  |  |  |  |  |  |  |  |  |  |  |  |  |  |  |  |  |  |  |  |  |  |  |  |  |  |  |  |  |  |  |  | MNP |  |
| *Trichys fasciculata* |  |  |  |  |  |  |  |  |  |  |  |  |  |  |  |  |  |  |  |  |  |  |  |  |  |  |  |  |  | KER |  |  | DVM |  |  |  |  |
| *Leopoldamys sabanus* |  |  |  |  |  |  |  |  |  |  |  |  |  |  |  |  |  |  |  |  |  |  |  |  |  |  |  |  | PSH |  |  |  |  |  |  |  |  |
| *Maxomys rajah* |  |  |  |  |  |  |  |  |  |  |  |  |  |  |  |  |  |  |  |  |  |  |  |  |  |  |  |  |  |  |  |  | DVM |  |  |  |  |
| *Rattus rattus* |  |  |  |  |  |  |  |  |  |  |  |  |  |  |  |  |  |  |  |  |  |  | DFS | DFN | RNF |  |  |  |  |  |  |  |  |  |  |  |  |
| *Rattus tiomanicus* |  |  |  |  |  |  |  |  |  |  |  |  |  |  |  |  |  |  |  |  |  |  |  |  |  |  |  |  | PSH |  |  |  |  |  |  |  |  |
| *Cricetomys emini* |  |  |  |  |  |  |  |  |  |  |  |  |  | INV |  | NNN | KRP | BIF | PWG |  |  |  | DFS | DFN |  |  |  |  |  |  |  |  |  |  |  |  |  |
| *Cricetomys gambianus* |  |  |  |  |  |  |  |  |  |  |  |  |  |  | UDZ |  |  |  |  | MIN | USZ | VIR |  |  |  |  |  |  |  |  |  |  |  |  |  |  |  |
| *Eliurus tanala* |  |  |  |  |  |  |  |  |  |  |  |  |  |  |  |  |  |  |  |  |  |  |  |  | RNF |  |  |  |  |  |  |  |  |  |  |  |  |
| *Nesomys rufus* |  |  |  |  |  |  |  |  |  |  |  |  |  |  |  |  |  |  |  |  |  |  |  |  | RNF |  |  |  |  |  |  |  |  |  |  |  |  |
| *Callosciurus erythraeus* |  |  |  |  |  |  |  |  |  |  |  |  |  |  |  |  |  |  |  |  |  |  |  |  |  |  |  |  |  |  |  |  | DVM |  |  |  |  |
| *Callosciurus finlaysonii* |  |  |  |  |  |  |  |  |  |  |  |  |  |  |  |  |  |  |  |  |  |  |  |  |  |  |  |  |  |  |  |  |  | TPH |  |  |  |
| *Dremomys rufigenis* |  |  |  |  |  |  |  |  |  |  |  |  |  |  |  |  |  |  |  |  |  |  |  |  |  |  |  |  |  |  |  |  |  |  |  |  | NAK |
| *Funisciurus anerythrus* |  |  |  |  |  |  |  |  |  |  |  |  |  |  |  |  | KRP |  |  |  |  |  |  |  |  |  |  |  |  |  |  |  |  |  |  |  |  |
| *Funisciurus carruthersi* |  |  |  |  |  |  |  |  |  |  |  |  |  |  |  |  |  | BIF |  |  |  |  |  |  |  |  |  |  |  |  |  |  |  |  |  |  |  |
| *Funisciurus isabella* |  |  |  |  |  |  |  |  |  |  |  |  |  |  |  |  |  |  | PWG |  |  |  | DFS |  |  |  |  |  |  |  |  |  |  |  |  |  |  |
| *Funisciurus leucogenys* |  |  |  |  |  |  |  |  |  |  |  |  |  |  |  |  | KRP |  |  |  |  |  |  |  |  |  |  |  |  |  |  |  |  |  |  |  |  |
| *Funisciurus pyrropus* |  |  |  |  |  |  |  |  |  |  |  |  |  |  |  |  | KRP |  |  | MIN |  |  | DFS | DFN |  |  |  |  |  |  |  |  |  |  |  |  |  |
| *Lariscus insignis* |  |  |  |  |  |  |  |  |  |  |  |  |  |  |  |  |  |  |  |  |  |  |  |  |  |  |  | BBS | PSH |  |  |  |  |  |  |  |  |
| *Menetes berdmorei* |  |  |  |  |  |  |  |  |  |  |  |  |  |  |  |  |  |  |  |  |  |  |  |  |  | HKK |  |  |  |  |  |  |  | TPH |  |  | NAK |
| *Paraxerus vexillarius* |  |  |  |  |  |  |  |  |  |  |  |  |  |  | UDZ |  |  |  |  |  | USZ |  |  |  |  |  |  |  |  |  |  |  |  |  |  |  |  |
| *Protoxerus stangeri* |  |  |  |  |  |  |  |  |  |  |  |  |  |  |  | NNN |  |  | PWG |  |  |  | DFS | DFN |  |  |  |  |  |  |  |  |  |  |  |  |  |
| *Rheithrosciurus macrotis* |  |  |  |  |  |  |  |  |  |  |  |  |  |  |  |  |  |  |  |  |  |  |  |  |  |  |  |  |  |  |  |  | DVM |  |  |  |  |
| *Sciurus aestuans* |  |  |  |  |  |  |  |  | CSN |  |  | MAN | |  |  |  |  |  |  |  |  |  |  |  |  |  |  |  |  |  |  |  |  |  |  |  |  |
| *Sciurus granatensis* |  |  |  |  |  | BCI |  |  |  |  |  |  |  |  |  |  |  |  |  |  |  |  |  |  |  |  |  |  |  |  |  |  |  |  |  |  |  |
| *Sciurus ignitus* |  |  |  |  |  |  |  |  |  |  | COU |  |  |  |  |  |  |  |  |  |  |  |  |  |  |  |  |  |  |  |  |  |  |  |  |  |  |
| *Sciurus igniventris* |  |  |  |  |  |  | YAS |  |  |  |  |  |  |  |  |  |  |  |  |  |  |  |  |  |  |  |  |  |  |  |  |  |  |  |  |  |  |
| *Sciurus spadiceus* |  |  |  |  |  |  |  |  |  | YAN |  |  |  |  |  |  |  |  |  |  |  |  |  |  |  |  |  |  |  |  |  |  |  |  |  |  |  |
| *Thryonomys swinderianus* |  |  |  |  |  |  |  |  |  |  |  |  |  |  | UDZ |  | KRP |  | PWG |  |  |  |  |  |  |  |  |  |  |  |  |  |  |  |  |  |  |
| *Tupaia belangeri* |  |  |  |  |  |  |  |  |  |  |  |  |  |  |  |  |  |  |  |  |  |  |  |  |  | HKK |  |  |  |  |  |  |  | TPH |  |  | NAK |
| *Tupaia glis* |  |  |  |  |  |  |  |  |  |  |  |  |  |  |  |  |  |  |  |  |  |  |  |  |  |  |  |  | PSH |  |  |  | DVM |  |  |  |  |
| TUBULIDENTATA |  |  |  |  |  |  |  |  |  |  |  |  |  |  |  |  |  |  |  |  |  |  |  |  |  |  |  |  |  |  |  |  |  |  |  |  |  |
| *Orycteropus afer* |  |  |  |  |  |  |  |  |  |  |  |  |  | INV |  | NNN |  |  | PWG |  |  |  | DFS |  |  |  |  |  |  |  |  |  |  |  |  |  |  |
|  |  |  |  |  |  |  |  |  |  |  |  |  |  |  |  |  |  |  |  |  |  |  |  |  |  |  |  |  |  |  |  |  |  |  |  |  |  |
