# Supplementary material for: Landscape-level human disturbance results in loss and contraction of mammalian populations in tropical forests
Source: PLoS Biol. 2025 Feb 13;23(2):e3002976. doi: 10.1371/journal.pbio.3002976 (PMC11825024; doi:10.1371/journal.pbio.3002976)
Supplement: S4 Table — Mean elevation (m.a.s.l.) of camera trap sites was sourced from the Global Biodiversity Information facility via package “rgbif” [1], mean annual precipitation (mm), and mean maximum temperature (°C) were sourced from the Worldclim historical monthly weather data [2], while the dominant landcover type was sourced from the MODIS Land Cover Type Yearly L3 Global 500 m. [3]. (DOCX) [file pbio.3002976.s009.docx]

S4 Table

List of the 37 areas included in the dataset with location, management type and environmental characteristics. Mean elevation (m.a.s.l.) of camera trap sites was sourced from the Global Biodiversity Information facility via package `rgbif`[1], mean annual precipitation (mm) and mean maximum temperature (°C) were sourced from the Worldclim historical monthly weather data[2], while the dominant landcover type was sourced from the MODIS Land Cover Type Yearly L3 Global 500 m.[3].

| **ID** | **Area name** | **Biogeographic region** | **Country** | **Management Regime** | **Mean elevation (m.a.s.l.)** | **Mean annual precipitation (mm)** | **Mean max temperature (°C)** | **Dominant landcover** |
| --- | --- | --- | --- | --- | --- | --- | --- | --- |
| BBS | Bukit Barisan | Indo-Malayan tropics | Indonesia | National Park | 122.93 | 2969.64 | 29.64 | Evergreen broadleaf forest |
| BCI | Barro Colorado | Neotropics | Panama | Nature Monument | 95.95 | 2472.87 | 31.05 | Evergreen broadleaf forest |
| BIF | Bwindi | Afrotropics | Uganda | National Park | 1906.58 | 1396.56 | 22.36 | Evergreen broadleaf forest |
| CAX | Caxiuanã | Neotropics | Brazil | National Forest | 43.48 | 2199.68 | 33.44 | Evergreen broadleaf forest |
| COU | Cocha Cashu | Neotropics | Perù | Biological Station | 350.39 | 2627.93 | 32.00 | Evergreen broadleaf forest |
| CSN | Central Suriname | Neotropics | Suriname | Nature Reserve | 45.70 | 2146.96 | 33.67 | Evergreen broadleaf forest |
| DFN | Somalomo | Afrotropics | Cameroon | Faunal Reserve | 599.31 | 1586.50 | 31.51 | Evergreen broadleaf forest |
| DFS | Djoum | Afrotropics | Cameroon | Faunal Reserve | 606.35 | 1585.24 | 31.05 | Evergreen broadleaf forest |
| DVM | Danum Valley | Indo-Malayan tropics | Malaysia | Conservation Area | 235.93 | 2560.83 | 30.45 | Evergreen broadleaf forest |
| GUR | Gurupi | Neotropics | Brazil | Biological Reserve | 143.19 | 1721.15 | 33.08 | Evergreen broadleaf forest |
| HKK | Huai Kha Khaeng | Indo-Malayan tropics | Thailand | Wildlife Sanctuary | 388.66 | 1425.52 | 35.18 | Evergreen/Deciduous broadleaf forest |
| INV | Ivindo NP | Afrotropics | Gabon | National Park | 432.39 | 1649.46 | 31.08 | Evergreen broadleaf forest |
| JAM | Jamari | Neotropics | Brazil | National Forest | 127.66 | 2369.55 | 33.47 | Evergreen broadleaf forest |
| KER | Kerinci Seblat | Indo-Malayan tropics | Indonesia | National Park | 615.88 | 2645.40 | 29.18 | Evergreen broadleaf forest |
| KRP | Korup | Afrotropics | Cameroon | National Park | 169.93 | 2578.08 | 31.55 | Evergreen broadleaf forest/Wetand |
| LEU | Gunung Leuser | Indo-Malayan tropics | Indonesia | National Park | 314.65 | 2966.20 | 30.57 | Evergreen broadleaf forest |
| LLA | Llanos | Neotropics | Colombia | Oil Palm Plantation | 254.21 | 2655.57 | 32.79 | Evergreen broadleaf forest/Woody savanna |
| MAS | Manaus | Neotropics | Brazil | Area of ecological interest | 106.15 | 2287.86 | 33.18 | Evergreen broadleaf forest |
| MIN | Minziro | Afrotropics | Tanzania | Nature Reserve | 1160.64 | 1328.08 | 27.13 | Evergreen broadleaf forest |
| MNP | Manas | Indo-Malayan tropics | India | National Park | 859.72 | 3977.81 | 27.21 | Evergreen broadleaf forest/Woody savanna |
| NAK | Nam Kading | Indo-Malayan tropics | Lao P.D.R. | Conservation Area | 659.12 | 2711.84 | 31.15 | Evergreen broadleaf forest |
| NNN | Nouabalé Ndoki | Afrotropics | Congo | National Park | 462.87 | 1630.74 | 31.47 | Evergreen broadleaf forest |
| PBN | Pau Brasil/Veracel | Neotropics | Brazil | National Park & private | 91.90 | 1419.72 | 30.80 | Evergreen broadleaf forest |
| PSH | Pasoh | Indo-Malayan tropics | Malaysia | Forest Reserve | 295.77 | 2220.00 | 31.39 | Evergreen broadleaf forest |
| PWG | PWG-CEB | Afrotropics | Gabon | Logging Concession | 341.61 | 1730.38 | 30.93 | Evergreen broadleaf forest |
| RKM | Rakhine | Indo-Malayan tropics | Myanmar | Non-Protected | 138.02 | 4163.10 | 34.17 | Evergreen broadleaf forest/Woody savanna |
| RNF | Ranomafana | Afrotropics | Madagascar | National Park | 111.80 | 1540.84 | 26.11 | Evergreen broadleaf forest |
| SHS | Sagaing Htamanthi | Indo-Malayan tropics | Myanmar | Wildlife Reserve | 207.24 | 2780.64 | 32.76 | Evergreen broadleaf forest |
| SUL | Sulawesi | Indo-Malayan tropics | Indonesia | National Park & Wildlife Sanctuary & Non-Protected | 645.77 | 2288.54 | 26.88 | Evergreen broadleaf forest |
| TDM | Terra do Meio | Neotropics | Brazil | Ecological Station | 175.97 | 1693.96 | 32.98 | Evergreen broadleaf forest |
| TPN | Ta Phraya NP | Indo-Malayan tropics | Thailand | National Park | 274.75 | 1112.82 | 33.95 | Evergreen broadleaf forest |
| UDZ | Udzungwa | Afrotropics | Tanzania | National Park | 1155.10 | 1369.55 | 25.87 | Evergreen broadleaf forest |
| UZS | Uzungwa scarp | Afrotropics | Tanzania | Nature Reserve | 1345.85 | 1413.02 | 26.29 | Evergreen broadleaf forest |
| VBA | Volcan Barva | Neotropics | Costa Rica | National Park | 705.43 | 3525.41 | 30.87 | Evergreen broadleaf forest |
| VIR | Virunga | Afrotropics | Rwanda | National Park | 3010.63 | 1544.34 | 19.79 | Evergreen broadleaf forest/Woody savanna |
| YAN | Yanachaga | Neotropics | Peru | National Park | 708.63 | 1972.05 | 30.99 | Evergreen broadleaf forest |
| YAS | Yasuni | Neotropics | Brazil | National Park | 192.40 | 1879.26 | 30.25 | Evergreen broadleaf forest |

**References**

1. Chamberlain S, Oldoni D, Barve V, Desmet P, Geffert L, Mcglinn D, et al. rgbif: Interface to the Global Biodiversity Information Facility API. 2023. Available: https://cran.r-project.org/web/packages/rgbif/index.html

2. Harris I, Jones P d., Osborn T j., Lister D h. Updated high-resolution grids of monthly climatic observations – the CRU TS3.10 Dataset. International Journal of Climatology. 2014;34: 623–642. doi:10.1002/joc.3711

3. Friedl M, Sulla-Menashe D. MCD12Q1 MODIS/Terra+Aqua Land Cover Type Yearly L3 Global 500m SIN Grid V061. NASA EOSDIS Land Processes DAAC; 2022. Available: https://doi.org/10.5067/MODIS/MCD12Q1.061
