# Supplementary material for: Landscape-level human disturbance results in loss and contraction of mammalian populations in tropical forests
Source: PLoS Biol. 2025 Feb 13;23(2):e3002976. doi: 10.1371/journal.pbio.3002976 (PMC11825024; doi:10.1371/journal.pbio.3002976)
Supplement: S5 Table — “Sampling effort (camera days)” is calculated as the total number of 24-h periods camera traps (CT) worked; “Season” attributes if data collection was conducted during the dry (period of reduced precipitation) or the wet season (period where most of the yearly rain is concentrated). (DOCX) [file pbio.3002976.s010.docx]

S5 Table

Details on sampling effort and design for the 37 areas included in the dataset. ‘Sampling effort (camera days)’ is calculated as the total number of 24-h periods camera traps (CT) worked; ‘Season’ attributes if data collection was conducted during the dry (period of reduced precipitation) or the wet season (period where most of the yearly rain is concentrated).

| **ID** | **Number of CT sites** | **Sampling effort (camera days)** | **Mean camera Days** | **Sampling year** | **Sampling period** | **Season** | **Average CT distance (km)** |
| --- | --- | --- | --- | --- | --- | --- | --- |
| BBS | 46 | 1058 | 23 | 2014 | Apr - Jul | DRY | 1.4 |
| BCI | 55 | 1760 | 32 | 2014/2015 | Dec - Mar | DRY | 1.4 |
| BIF | 59 | 1475 | 25 | 2014 | May - Jul | DRY | 1 |
| CAX | 58 | 1914 | 33 | 2014 | Aug - Oct | DRY | 1.4 |
| COU | 59 | 1711 | 29 | 2014 | Jun - Aug | DRY | 1.5 |
| CSN | 50 | 1500 | 30 | 2014 | Sep - Dec | DRY | 1.4 |
| DFN | 32 | 1056 | 32 | 2015 | Nov-May | DRY | 1.9 |
| DFS | 37 | 1080 | 30 | 2017 | May-Jun | WET | 1.8 |
| DVM | 42 | 1260 | 30 | 2015 | Jun - Jul | DRY | 1 |
| GUR | 57 | 1824 | 32 | 2016 | Oct - Nov | DRY | 1.4 |
| HKK | 65 | 2015 | 31 | 2010/2011 | Nov - Mar | DRY | 1.2 |
| INV | 35 | 1400 | 40 | 2019 | May - Jun | DRY | 1 |
| JAM | 53 | 1643 | 31 | 2017 | Sept - Nov | DRY | 1.7 |
| KER | 48 | 1344 | 28 | 2014 | May - Aug | DRY | 1.5 |
| KRP | 59 | 1770 | 30 | 2014 | Jan - Apr | DRY | 1.4 |
| LEU | 54 | 2160 | 40 | 2014 | Jan - Mar | DRY | 1.5 |
| LLA | 45 | 1350 | 30 | 2014/2015 | Sept - Jan | DRY | 2.2 |
| MAS | 59 | 1888 | 32 | 2010 | Jul - Nov | DRY | 1.4 |
| MIN | 68 | 2244 | 33 | 2018 | Oct - Dec | DRY | 1.1 |
| MNP | 60 | 1860 | 31 | 2016/2017 | Dec - Jan | DRY | 1.6 |
| NAK | 56 | 1848 | 33 | 2014/2015 | Oct - Jan | DRY | 1.4 |
| NNN | 53 | 2226 | 42 | 2014/2015 | Dec - Mar | DRY | 1.4 |
| PBN | 57 | 1767 | 31 | 2018 | Aug - Sep | DRY | 1.4 |
| PSH | 57 | 1653 | 29 | 2014 | May - Jul | DRY | 1.3 |
| PWG | 54 | 1512 | 28 | 2018/2019 | Sep - Jan | WET | 1.4 |
| RKM | 60 | 1980 | 33 | 2017/2018 | Nov - Feb | DRY | 1 |
| RNF | 59 | 1593 | 27 | 2013/2014 | Nov - Feb | DRY | 1.4 |
| SHS | 61 | 1952 | 32 | 2017/2018 | Dec - Mar | DRY | 1.1 |
| SUL | 49 | 1519 | 31 | 2018 | Apr - Jul | DRY | 5 |
| TDM | 58 | 1914 | 33 | 2016 | Jun - Jul | DRY | 1.3 |
| TPN | 62 | 1488 | 24 | 2010 | Nov - Feb | DRY | 1.5 |
| UDZ | 59 | 1829 | 31 | 2014 | Jul - Nov | DRY | 1.4 |
| UZS | 60 | 1860 | 31 | 2017 | Jul - Nov | DRY | 1.5 |
| VBA | 59 | 1770 | 30 | 2014 | Jan - Apr | DRY | 1.4 |
| VIR | 58 | 1914 | 33 | 2015 | Jul - Oct | DRY | 1.4 |
| YAN | 58 | 1624 | 28 | 2014 | Jul - Sept | DRY | 1.4 |
| YAS | 60 | 2280 | 38 | 2014 | Feb - May | DRY | 1.4 |
